# Assessing Barrier Function in Psoriasis and Cornification Models of Artificial Skin Using Non‐Invasive Impedance Spectroscopy

**DOI:** 10.1002/advs.202400111

**Published:** 2024-07-12

**Authors:** Jaehwan Ahn, Yoon Sung Nam

**Affiliations:** ^1^ Department of Materials Science and Engineering Korea Advanced Institute of Science and Technology 291 Daehak‐ro, Yuseong‐gu Daejeon 34141 Republic of Korea; ^2^ Department of Biological Sciences Korea Advanced Institute of Science and Technology 291 Daehak‐ro, Yuseong‐gu Daejeon 34141 Republic of Korea

**Keywords:** impedance spectroscopy, psoriasis, reconstructed epidermal equivalents, skin barrier, stratum corneum

## Abstract

Reconstructed epidermal equivalents (REEs) consist of two distinct cell layers – the stratum corneum (SC) and the keratinocyte layer (KL). The interplay of these layers is particularly crucial in pruritic inflammatory disorders, like psoriasis, where a defective SC barrier is associated with immune dysregulation. However, independent evaluation of the skin barrier function of the SC and KL in REEs is highly challenging because of the lack of quantitative methodologies that do not disrupt the counter layer. Here, a non‐invasive impedance spectroscopy technique is introduced for dissecting the distinct contributions of the SC and KL to overall skin barrier function without disrupting the structure. These findings, inferred from the impedance spectra, highlight the individual barrier resistances and maturation levels of each layer. Using an equivalent circuit model, a correlation between impedance parameters and specific skin layers, offering insights beyond traditional impedance methods that address full‐thickness skin only is established. This approach successfully detects subtle changes, such as increased paracellular permeability due to mild irritants and the characterization of an immature SC in psoriatic models. This research has significant implications, paving the way for detailed mechanistic investigations and fostering the development of therapies for skin irritation and inflammatory disorders.

## Graphical abstract

Electrical impedance spectroscopy enables non‐invasive, independent evaluation of barrier functions within different cellular layers of reconstructed epidermal equivalents. This technique provides a detailed analysis of both the stratum corneum and keratinocyte layer. Particularly, it can detect changes in barrier function caused by skin irritants and can be useful in understanding the progression of inflammatory disorders, such as psoriasis.

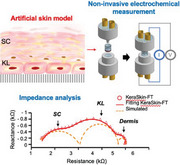

## 1 Introduction

Reconstructed epidermal equivalents (REEs) are emerging as an alternative model to animal and clinical studies due to their reduced inter‐individual variability, lower costs, and fewer ethical issues.^[^ ^1^, ^2^, ^3^ ^]^ REEs consist of the upper stratum corneum (SC) and a proliferating keratinocyte layer (KL) on a porous polymer membrane. Cell differentiation replaces the plasma membrane of keratinocytes with cornified (non‐hydrated) lipid envelopes, the cells flattening on the air–liquid interface.^[^ ^4^, ^5^ ^]^ With an intercellular lipid matrix, the SC layer forms a multilayer structure of flattened cells, which creates a strong lipophilic barrier that limits the transdermal permeation of molecules, whereas the basal layer consists of viable keratinocytes.^[^ ^6^ ^]^ Owing to their structural and functional similarities to the human skin, REEs can be used for a wide range of analyses, including skin irritation, regeneration, elasticity, and toxicological screening.^[^ ^7^, ^8^, ^9^, ^10^ ^]^

The main drawback of REEs is the low reproducibility of SC formation compared to *ex vivo* skin.^[^ ^11^ ^]^ Unlike *ex vivo* human and animal skin, the structural changes in REEs are dynamic and can be unpredictable during incubation. Keratinocytes near the air–liquid interface can become anucleate squamous cells, making the SC layer thicker and more lipophilic.^[^ ^12^ ^]^ As the hydrophobicity of solutes greatly affects their skin permission route, differences in SC maturation can lead to inconsistent experimental results.^[^ ^13^, ^14^ ^]^ Despite these limitations, many studies using the REE model have not justified the maturation effect on the skin barrier function before conducting skin tests.^[^ ^15^, ^16^, ^17^ ^]^

Additionally, assessing the degree of SC differentiation from keratinocytes is important because immune‐related skin diseases, such as psoriasis and atopic dermatitis, have terminal differentiation defects in keratinocytes and immunological abnormalities.^[^ ^18^ ^]^ Although the precise mechanism is not fully understood, a combination of environmental and genetic factors can disrupt the SC barrier function, allowing for the skin penetration of irritants and allergens to aggravate pruritic skin conditions.^[^ ^19^, ^20^ ^]^ Some chemical ingredients in topical products, including surfactants, glycols, and solvents, can affect the skin barrier function by carrying biologically active substances into the deeper layers of the skin.^[^ ^21^ ^]^ Accordingly, a precise and intertemporal assessment of the contributions of the two distinct skin barrier functions is required to validate REEs as an in vitro skin model. Also, precise analysis of the SC barrier function considering the degree of SC differentiation is important for evaluating the in vitro efficiency and safety of personal care and skin‐related pharmaceutical products.^[^ ^22^ ^]^

Various analytical techniques have been employed to investigate the integrity of REEs, including transepithelial electrochemical resistance (TEER), dye penetration analyses, immunohistochemistry, and vibrational spectroscopic analysis.^[^ ^23^ ^]^ However, none of these meet three conditions simultaneously – that is, they are non‐invasive, quantitative, and can distinguish the barrier function of the SC from the epidermal layer.^[^ ^24^ ^]^ Previous quantitative techniques, such as TEER and dye penetration testing, have been unable to distinguish between the barrier functions of the SC and the viable epidermis.^[^ ^24^ ^]^ In contrast, immunohistochemistry combined with tracers provides the most detailed information about how barriers work and where certain tracers are. However, this is a semi‐quantitative method that requires destructive sample preparation protocols. It can only be used to look at specific points in time in tissue samples taken from skin biopsies and at quantitative data about flux.^[^ ^25^, ^26^, ^27^, ^28^ ^]^ Finally, vibrational spectroscopy techniques, such as Raman spectroscopy and Fourier transform infrared (FTIR) spectroscopy, are useful tools for studying the molecular lipid structure within the SC to evaluate the skin barrier of in vitro skin equivalents.^[^ ^29^, ^30^ ^]^ However, skin complexity can result in highly complex Raman or FTIR spectra, obscuring drug‐related signals and making it difficult to understand the experimental results because they are not direct measurements of the barrier function.^[^ ^31^ ^]^

Electrochemical impedance spectroscopy (EIS) has been used to assess the barrier function of various epithelial monolayers, including oral epithelium and skin.^[^ ^32^, ^33^, ^34^, ^35^, ^36^, ^37^ ^]^ Unlike TEER, which provides a single value, EIS offers a frequency‐dependent impedance spectrum with identifiable relaxation peaks. Analyzing these spectra using an equivalent circuit model helps simulate the epithelial response to alternating current. Typically, an RC circuit model, pairing a resistor for the extracellular matrix with a capacitor for the cellular membrane, produces a Cole impedance model with a semicircular arc matching the biological impedance spectrum.^[^ ^32^, ^38^ ^]^ Yamamoto and Yamamoto (1976) suggested that reconstituted human epidermis (RHE) impedance consists of two distinct electrical barriers for the SC and the viable layer (VL), grounded in the observed differences in their average resistivities and dielectric constants. To represent these two barriers, they proposed an equivalent circuit model consisting of two parallel RC circuits, aligning with in vivo skin and isolated epidermis characteristics. While EIS has mathematically been shown to detect variations in the skin barrier, discerning the specific influences on the VL and SC still remained a challenge.

Given the complex structure of the cellular membrane, it behaves more like a leaky capacitor rather than an ideal one.^[^ ^34^ ^]^ Thus, introducing a constant phase element (CPE) is necessary for accurately depicting its dynamic electrical characteristics.^[^ ^39^ ^]^ The R‐CPE model, integrating a resistor with the CPE, is commonly used in EIS studies of excised skin.^[^ ^35^, ^40^, ^41^, ^42^ ^]^ Groeber et al. (2015) used the R‐CPE model to assess RHE barrier function, finding impedance spectra of fully matured RHE comparable to human epidermal biopsies and suggesting capacitance and ohmic resistance as metrics for identifying skin irritants.^[^ ^35^ ^]^ However, they found the SC resistance significantly higher than the VL's, without experimental evidence of two distinct layers. Kiesewetter et al. (2019) also applied CPEs in their model to analyze the impedance spectra of wounded RHE, focusing on wound healing and barrier function restoration.^[^ ^41^ ^]^ Beier et al. (2022) used CPEs to describe the capacitive properties of the vital epithelium and SC, evaluating tight junctions and barrier integrity, particularly in claudin protein studies.^[^ ^42^ ^]^ Despite using CPEs, these studies had different experimental conditions, including frequency range, electrodes, and analyzed samples. Groeber et al. and Kiesewetter et al. used complex circuits with multiple CPEs and RC circuits, while Kiesewetter et al. added parallel resistance for wound areas, and Beier et al. utilized a simpler model with a single CPE, focusing on the capacitive properties of specific epithelial layers.

EIS can also monitor impedance changes indicative of SC barrier degradation, common in inflammatory skin diseases like allergic and atopic dermatitis (AD).^[^ ^43^, ^44^ ^]^ Rinaldi et al. (2021) demonstrated EIS's potential as a non‐invasive diagnostic tool for AD, improving diagnostic accuracy, monitoring disease progression, and informing treatment strategies. Recent studies have independently evaluated the barrier functions of the SC and VL using EIS. Mannweiler et al. (2021) identified distinct relaxation frequencies for the SC (500 Hz to 1 MHz) and VL (10 Hz to 100 Hz), indicating their separate barrier properties.^[^ ^45^ ^]^ They observed layer‐specific frequency shifts when applying chemicals and nanoparticles to the RHE, examining impedance spectra dynamics during hyperosmolar electrolyte penetration. This analysis could help detect barrier dysfunctions associated with tight junction proteins.

In this study, we investigated the barrier function of the SC layer and viable epidermis in complex REEs by employing EIS. To quantitatively evaluate the electrochemical changes in commercial REEs affected by SC maturation, we designed and fabricated an EIS system for REEs. We also compared the impedance spectra of various skin models, such as REEs manufactured by different companies, the full‐thickness skin model, mouse skin, and porcine skin, to ensure the reliability of the measurement system. Impedance spectroscopy showed the existence of two distinct layers only in REEs, the relative impedance components of which could be determined by equivalent circuit modeling and impedance simulation because of the different electrochemical properties of corneocytes in the SC layer and keratinocytes in the epidermis. The correlation between the impedance variable and physiological characteristics, such as the SC thickness and keratinocyte differentiation, was determined using histological staining. In addition, two impedance methods were introduced to assess the damage caused by REEs to sodium dodecyl sulfate (SDS), a common model skin irritant. Time‐lapse impedance spectroscopy revealed an increase in paracellular permeability after low‐concentration of SDS treatment with equivalent circuit analysis and BioZsim simulations. Subsequently, electrochemical changes of two different layers of REEs in established psoriasis skin models and healthy skin models were compared.

## 2 Results and Discussion

### 2.1 Method Overview

**Figure 1:**
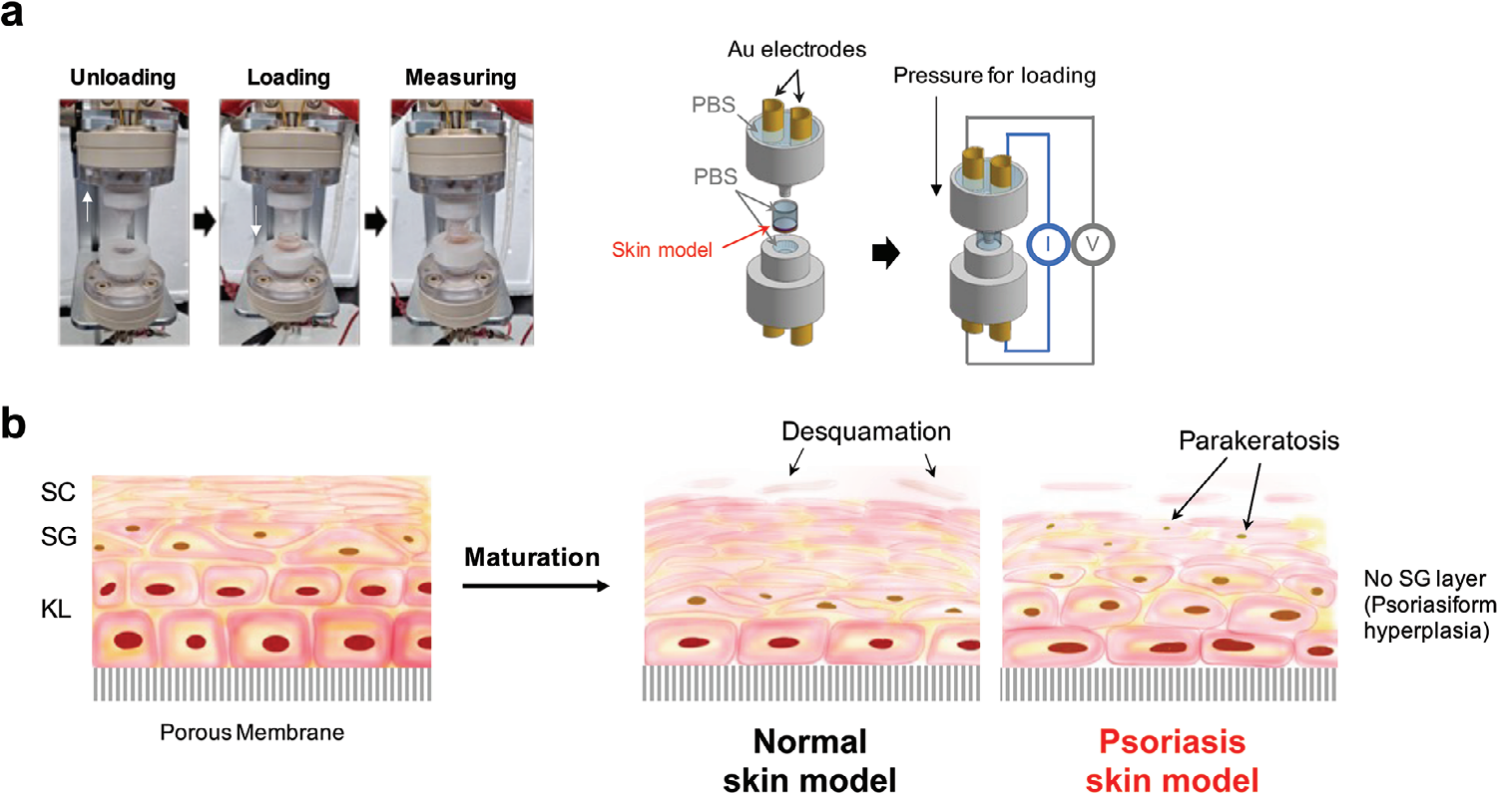
a) A digital photograph and a schematic description of the custom‐built impedance analysis system for REEs. b) In a healthy skin model, keratinocytes along the air–liquid interface transform into squamous cells. Proliferative keratinocytes undergo enucleation and dehydration in the stratum granulosum (SG), which induces thickening, lyophilization, and desquamation of the stratum corneum (SC). KL stands for the keratinocyte layer. In a psoriasis model, incomplete maturation of keratinocytes with abnormal retention of nuclei (parakeratosis) can exist in the SC.

Our custom‐designed four‐electrode impedance analysis system, depicted in **Figure** **1**a, offers significant advantages for the study of REEs over traditional two‐electrode systems. Its capacity to isolate current and voltage inputs/outputs enables the avoidance of electrode transfer and contact impedance effects, thereby ensuring more accurate measurements.^[^ ^46^ ^]^ An REE was cultured on a semipermeable filter (10 mm in diameter) in a chamber with a 4 mm opening. The chamber was plugged into the system's lower chamber and filled with phosphate buffered saline (PBS). A cross‐sectional view of REEs exhibiting normal SC maturation and aberrant SC maturation in a psoriasis model is illustrated in Figure 1b.

The REE model consists of a polyethylene terephthalate membrane with 0.4 µm pores, underlain by a KL, a stratum granulosum (SG) layer, and an SC layer. These cellular layers are collectively ≈100 µm thick, closely approximating the thickness of native skin, with the SC layer ranging from 10–30 µm in thickness. Due to distinct differences in relative permittivity between the KL and SC layers, impedance fitting analysis allows for the discrete assessment of each layer's electrochemical properties.^[^ ^47^ ^]^ The psoriatic model is characterized by a depressed SG layer and retention of nuclei in the superficial keratinocytes, indicators of psoriasiform hyperplasia and parakeratosis.^[^ ^48^ ^]^

Skin impedance analysis has been used to evaluate the integrity of the completely matured epidermis. However, the previous study did not differentiate between the SC and KL layers, treating the epidermis as a singular electrical barrier. It focused on the effects of mechanical and chemical damage on the epidermis but not on biological barrier dysfunction, such as that caused by undifferentiated SC and immune dysregulation.^[^ ^49^ ^]^ On the contrary, our method was designed to determine the distinct electrochemical properties of the SC and KL layers, allowing for monitoring barrier degradation in each layer caused by irritant penetration. Furthermore, our system can detect the electrochemical changes of immature SC and KL layers induced by immune‐related disturbances in a psoriatic skin model.

### 2.2 Impedance Characteristics of REEs During SC Maturation

**Figure 2:**
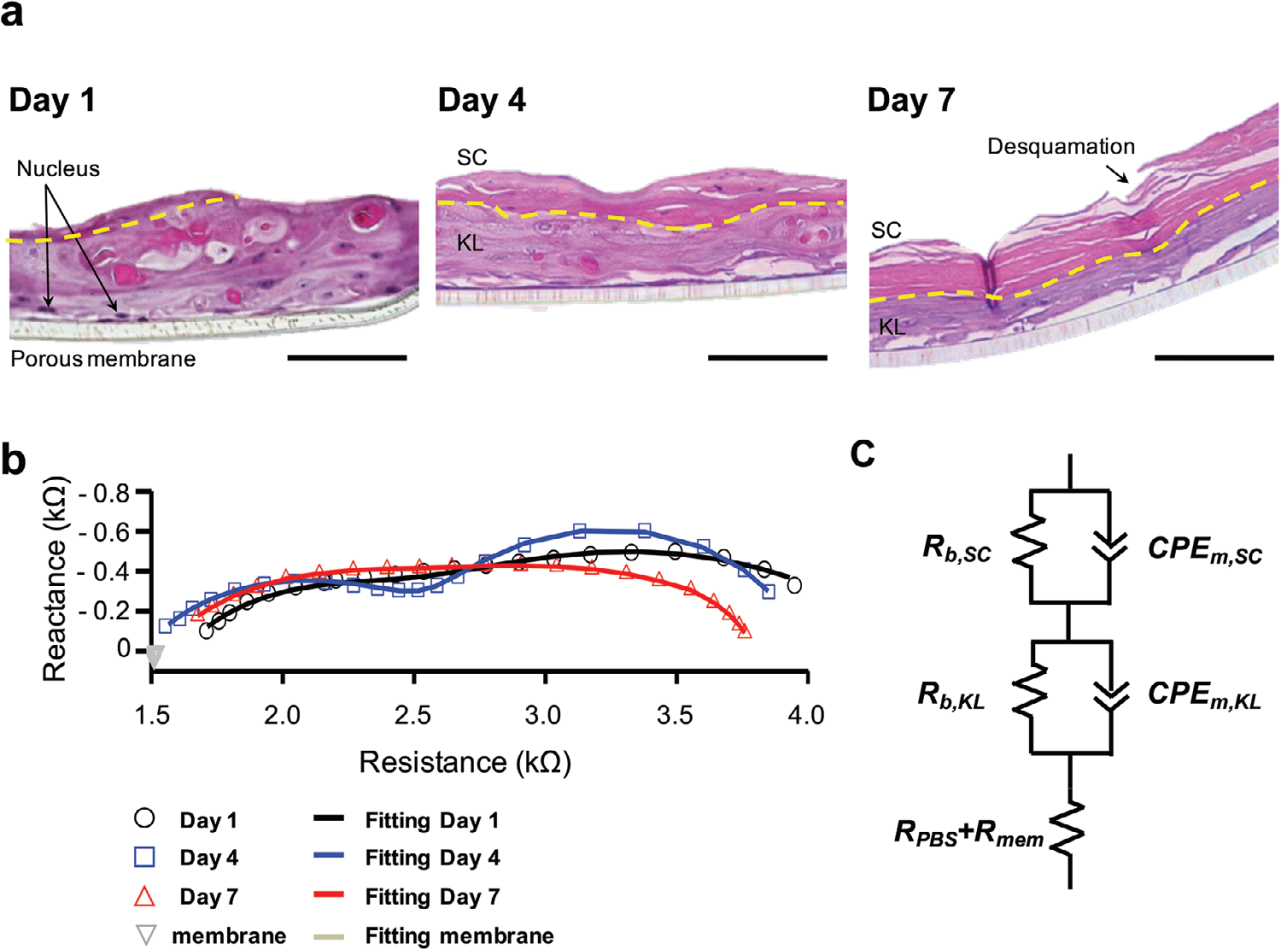
a) H&E‐stained histopathological images of the REEs (scale bar = 100 µm). b) Nyquist plots of the REEs on days 1, 4, and 7 of incubation with the frequency range of 10 to 10^5^ Hz, where symbols indicate the measurement data and curved lines indicate the fitted data. c) shows the equivalent circuit model for the REE, where *R_b,KL_* and *R_b,SC_* denote the barrier resistances of the KL and SC layer; *CPE_m,KL_*, and *CPE_m,SC_* denote the constant phase elements of the plasma membrane of two‐skin layers, and *R_PBS_ + R_mem_* denotes the resistance of the PBS and the porous membrane.

REEs were cultured at the air‐medium interface for 7 days to form the SC layer according to the previous reports on the progression of epidermal differentiation and associated electrochemical changes.^[^ ^50^, ^51^, ^52^ ^]^ Hematoxylin and eosin (H&E) staining revealed the developmental stages of the REEs on 1, 4, and 7 days, particularly noting the emergence of the SC layer, as shown in **Figure** **2**a. On the first day, the SC layer was incomplete, but by day 4, keratinocyte maturation became apparent, including flattening and loss of nuclei – a critical step in their maturation process. In the keratinocyte life cycle, as new cells form, they push the premature ones upwards, eventually peeling off the outermost dead cells, a process known as desquamation.^[^ ^53^ ^]^ By the seventh day, a marked decrease in cell viability was observed, dropping to 39.1% as shown in Figure S1 (Supporting Information), possibly due to the differentiation of viable keratinocytes into non‐viable corneocytes over time. This process includes semi‐apoptotic events characterized by the loss of cell organelles and nuclei.^[^ ^54^ ^]^ In native skin, the basal layer's production of new cells propels the formation of prickle cells, which move outward until they reach the granular layer, a process described by Murphrey et al.^[^ ^5^ ^]^ However, the REE model exhibits a reduction in viability over time, attributed to a finite reserve of basal keratinocytes, which limits the formation and upward movement of prickle cells.

The Nyquist plots in Figure 2b show the impedance spectra for each group of REEs, alongside their equivalent circuits, highlighting the electrochemical changes that occur during the maturation of the REEs. On day 4, two distinct semicircles in the plots indicate separate electrochemical properties within the REEs, in contrast to the merging semicircles observed on days 1 and 7. Typically, the electrochemical behaviors of biological cells can be modeled using a simplified circuit that pairs cell barrier resistance (R) with cell membrane capacitance (C), described by the single‐dispersion Cole impedance model, which predicts a singular semicircle on a Nyquist plot.^[^ ^55^ ^]^

However, the REEs' impedance spectra suggest a better fit with a more complex double‐dispersion Cole impedance model. This expanded model includes two sets of parallel R‐CPE (resistance and constant phase element) pairs to account for the imperfect capacitive nature of the cell lipid layers, arranged in series (Figure 2c). This model is substantiated by the distribution of relaxation times caused by the REEs’ electrochemical and structural heterogeneity, and by variances in relative permittivity, size, shape, and integrity of the cell membranes, as reflected by the CPE measurements.^[^ ^38^, ^55^ ^]^ The constant relaxation frequencies for both layers, irrespective of time, indicate the two distinct barrier components.^[^ ^43^ ^]^ The disparity in the impedance of the SC layer and the deeper tissues in REEs necessitates the dual R‐CPE model.^[^ ^52^, ^55^ ^]^ By conducting impedance analyses on skin models with varying numbers of layers, we aimed to discern the electrochemical signatures of each layer's barrier.

### 2.3 Comparison of Impedance Characteristics of Skin Equivalents

**Figure 3:**
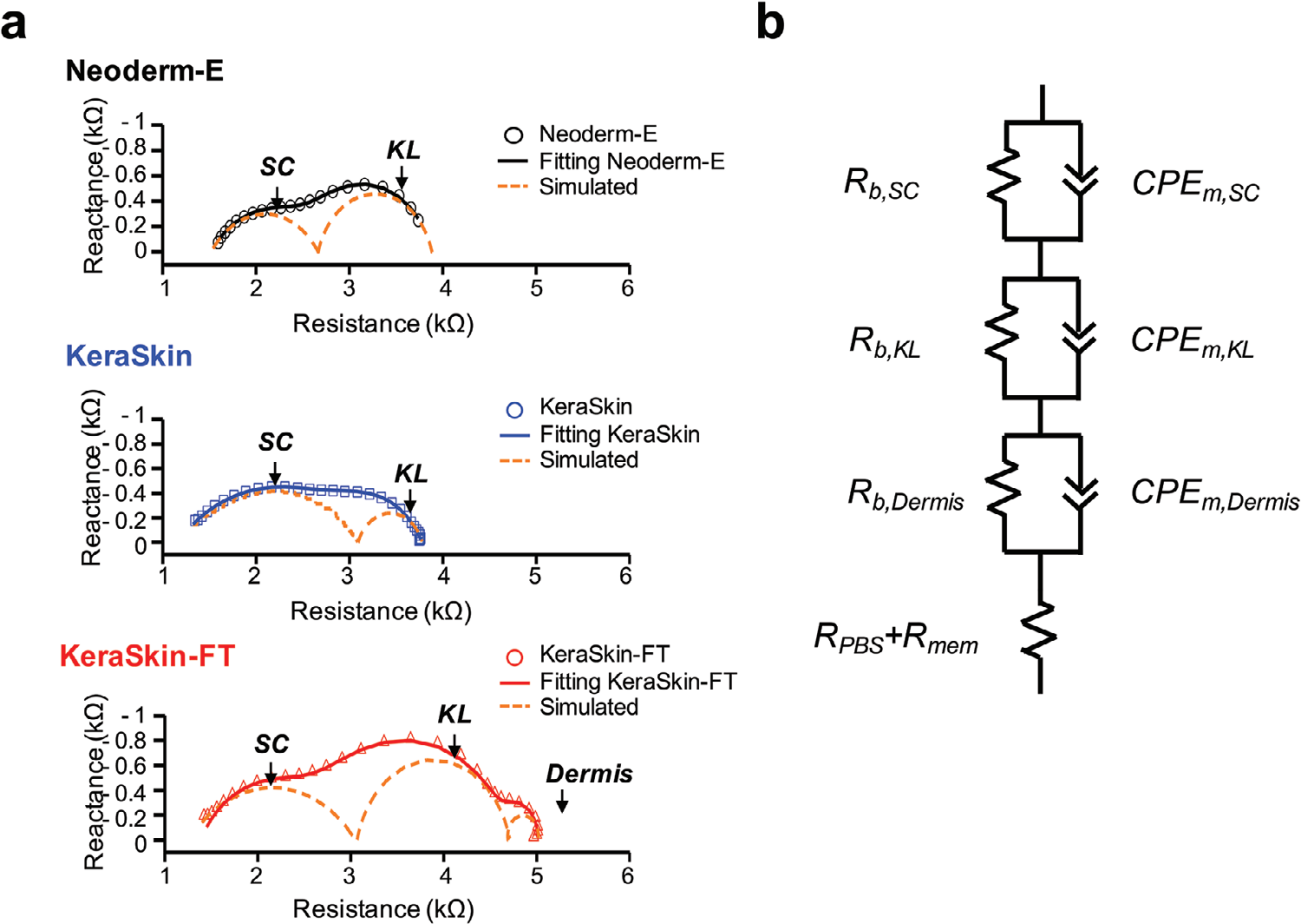
a) Nyquist plots of three different types of human skin models (Neoderm‐E, KeraSkin, and KeraSkin‐FT) on day 4 of incubation, with frequency ranges ranging from 10^−1^ to 10^5^ Hz, where symbols indicate the measurement data, curved lines indicate the fitted data and orange dot lines indicate the simulated contributions of each layer. b) shows the equivalent circuit for the full‐thickness skin model, Keraskin‐FT, where *R_b,Dermis_, R_b,KL_*, and *R_b,SC_* are the barrier resistances of the dermis, KL, and SC layer, respectively; *CPE_m,Dermis_, CPE_m,KL_*, and *CPE_m,SC_* are constant phase elements of the plasma membrane of each skin layers, respectively, and *R_PBS_ + R_mem_* is the resistance of the PBS and the porous membrane.

Impedance spectra for two REE models, Neoderm‐E and KeraSkin, along with a full‐thickness skin model, KeraSkin‐FT, are shown in Nyquist plots (**Figure** **3**a) with the equivalent circuit of KeraSkin‐FT (Figure 3b). KeraSkin's impedance agreed with an equivalent circuit characterized by a series of two R‐CPEs. In contrast, the impedance spectrum of KeraSkin‐FT corresponded to a circuit with three such series of components. The impedance fitting process elucidated the contribution of each distinct layer, visualized as overlapping semicircles delineated by an orange dashed line in the Nyquist plots. Specifically, KeraSkin‐FT exhibited a unique, low‐frequency semi‐circle, absent in other REE models, attributed to the dermal layer's fibroblasts (Figure S2, Supporting Information), and is hence labeled “Dermis.” The high‐frequency semicircle in the Nyquist plot was markedly more depressed, with significantly lower “*n*” values than seen in other spectra, indicative of non‐ideal capacitive behavior. This behavior suggests anomalies such as increased roughness, leakage capacitance, or non‐uniform current distribution, which are symptomatic of a compromised plasma membrane's ion‐charging capacity, compared to that of a healthy cell.^[^ ^16^ ^]^ Corneocytes, devoid of phospholipids and thus unable to retain ions, exhibited lower water content (15–30%) than viable cells (70%).^[^ ^56^ ^]^ Consequently, the SC layer had lower relative permittivity and capacitance than the KL, especially when considering the negligible differences in plasma membrane area and intercellular distances.^[^ ^52^, ^57^, ^58^ ^]^ This disparity allowed us to correlate the left and right semi‐circuits in the Nyquist plots with the electrochemical properties of SC and KL, labeling them as “SC” and “KL,” respectively. Furthermore, the “*n*” value associated with SC's CPE was lower than that of other viable cell layers, signifying a less stable corneocyte membrane in terms of ionic displacement, akin to an imperfect capacitor (Figure S3, Supporting Information).

We also analyzed the impedance spectra of full‐thickness mouse skin, including epidermis and dermis layers separated using dispase II. The impedance range for mouse skin was notably broader by a factor of fifteen than that of the REEs, attributed to the presence of adipose tissue in the mouse skin (Figure S4A, Supporting Information).^[^ ^59^ ^]^ Moreover, the impedance values for the separated dermis were lower, reflecting its composition of a predominantly hydrated cellular matrix with sparse fibroblasts (Figure S4B, Supporting Information). The epidermis, once isolated from the mouse dermis, was modeled using two different equivalent circuits to account for varied electrochemical barriers (Figure S4C, Supporting Information). Our analysis favors “model 2,” described in Figure 2c, over the simpler ‘model 1′ from prior literature (Figure S4D, Supporting Information).^[^ ^60^ ^]^ The full‐thickness mouse skin, with its more complex structure, presents an impedance too substantial in the adipose and undigested dermis layers to discern the contributions from SC and KL. Nonetheless, our impedance measurements successfully differentiated the electrochemical barriers of each cell layer within various skin models, including ex vivo mouse skin. Accordingly, through the application of a REE equivalent circuit model and simulation analysis, we determined the impact of model parameters on the impedance profiles, thereby enhancing our understanding of skin electrochemical properties.

### 2.4 Simulation Analysis of an Equivalent Circuit Model for REEs

**Figure 4:**
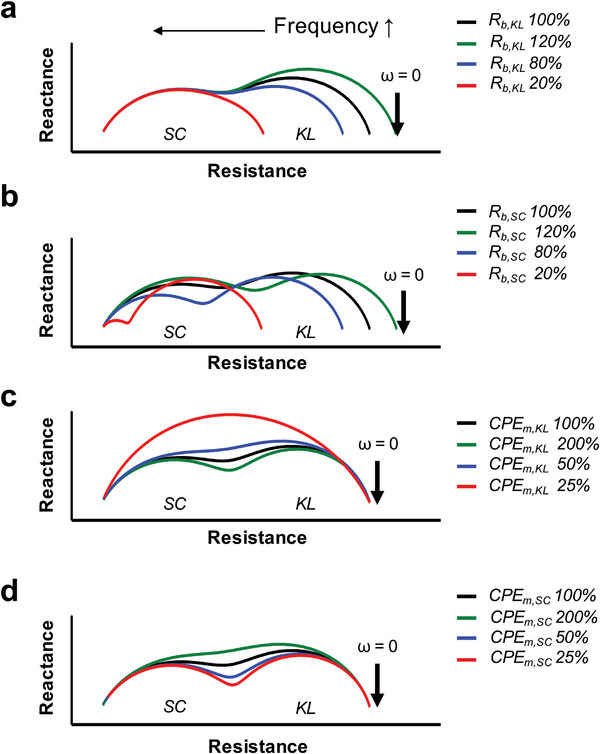
Computational simulation results using ZView of *R_b,KL_* a), *R_b,SC_* b), *CPE_m,KL_* c), and *CPE_m,SC_* d) using the equivalent model shown in Figure 2c. The impedance fitting data of the REEs on day 4 was used to draw the control curve (black).

To investigate alterations in the impedance spectra, we systematically manipulated the relative values of individual parameters, as depicted in **Figure** **4**. The fitted data of REEs on day 4 were used as a reference baseline (indicated by the black line). The changes in impedance parameters associated with the left and right semi‐circular arcs denoted as SC and KL, respectively, exhibit discernible variations in response to adjustments in *R_b,SC_* and *R_b,KL_* as presented in Figure 4a,b. Furthermore, we observed that modifications in the total resistance in series (at *ω* = 0) are directly influenced by changes in the R values, while alterations in *CPE_m,KL_* and *CPE_m,SC_* govern the separation of overlapping semicircles, as illustrated in Figure 4c,d. When the difference in CPE values between the two layers becomes substantial, the two impedance barriers become distinguishable. Each layer exhibits distinct relaxation characteristics when the difference in capacitance between these layers reaches a significant threshold, as previously reported by Manouras et al.^[^ ^61^ ^]^ Consequently, the disparity in the electrochemical charge quantity stored on the plasma membrane within each layer dictates the emergence of well‐defined semicircles in the Nyquist plot. Following our fitting analysis, we were able to precisely determine the barrier resistance values (*R_b,KL_* and *R_b,SC_*) as well as the constant phase elements of the plasma membrane (*CPE_m,KL_* and *CPE_m,SC_*) for each layer, contributing valuable insights to our research.

**Figure 5:**
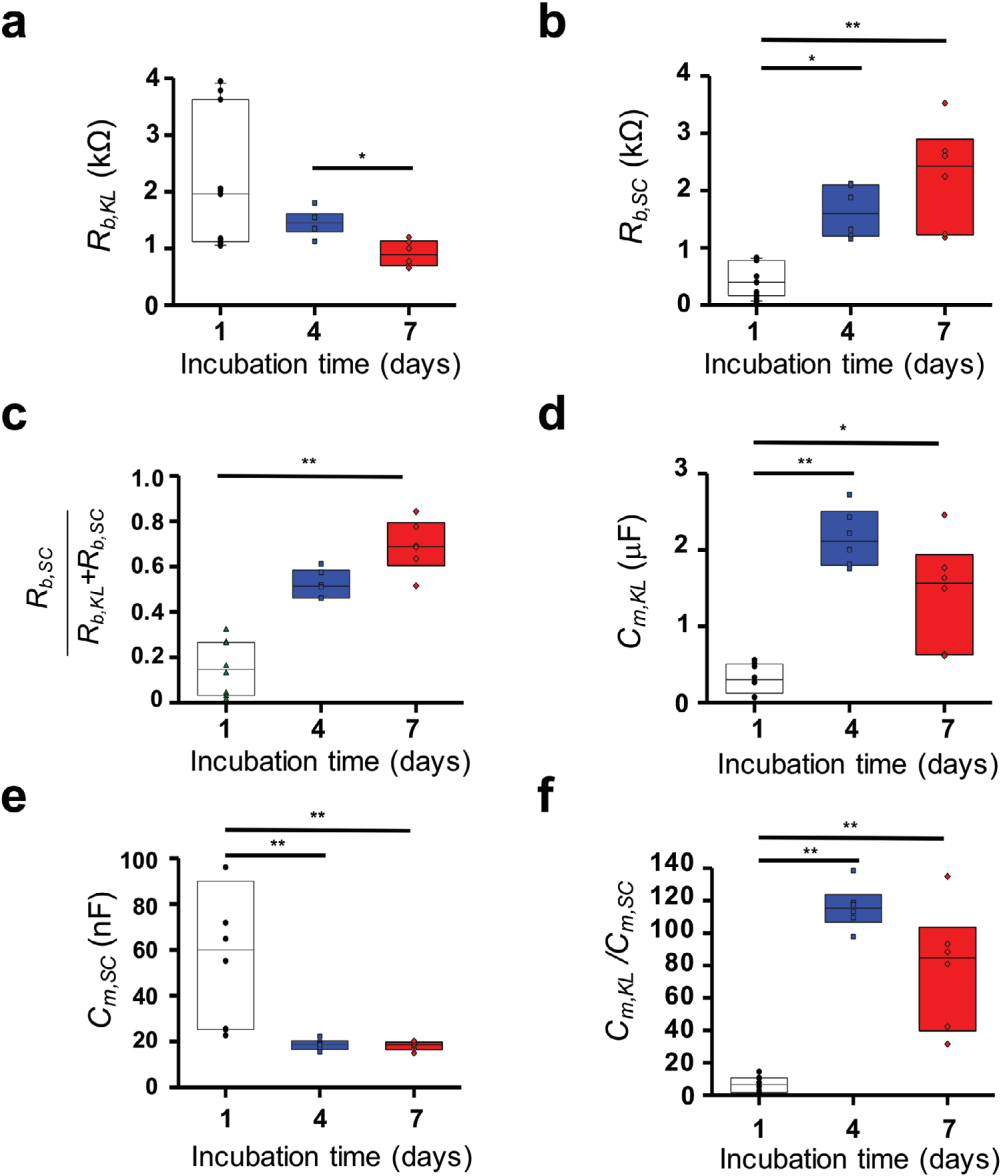
Impedance parameters: *R_b,KL_* a), *R_b,SC_* of the REEs b), the electrochemical contribution by the proposed equation c), *C_m,KL_* (d), *C_m,SC,_* from Equation (2) e), and *C_m,KL_/C_m,SC_* f). ^*^ *p *< 0.05 and ^**^ *p *< 0.01. The median marks the mid‐point of the box. The down and up sides of the box indicate the lower and upper quartiles, respectively.

The cellular components within each layer of the REEs were characterized using a mathematical model represented by Equation (2) in terms of barrier resistance (*R_b_*) and plasma membrane capacitance (*C_m_*) (**Figure** **5**). A gradual decrease in *R_b,KL_* coupled with an increase in *R_b.SC_* was observed as the incubation time progressed (Figure 5a,b). Our findings agree with the thickness increment observed in the H&E image in Figure 2a (Figure S5, Supporting Information). To gain further insight, we calculated the ratio of resistance associated with the SC layer to the overall REE resistance, denoted as *R_b,SC,_*/(*R_b,KL_* +*R_b,SC_*) (Figure 5c), and our results unveiled a significant increase from 14.1% on day 1 to 68.7% on day 7, signifying the corneous layer's dominance within the REE. On day 4, we observed a noteworthy change in *C_m_* values, with *C_m,KL_* increasing from 319.5 to 2157.3 nF, while *C_m,SC_* decreased from 65.2 to 18.6 nF, as presented in Figure 5d,e. Correspondingly, the ratios of *C_m,KL_*/*C_m,SC_* exhibited a distinctive pattern, escalating from 6.7 on day 1 to 115.8 on day 4, followed by a decrease to 78.5 on day 7 (Figure 5f). This high ratio of *C_m,KL_*/*C_m,SC_*, discernible through the presence of two well‐defined semicircles in the Nyquist plot, indicates the development of a distinct electrochemical profile within the KL layer.^[^ ^52^, ^61^ ^]^

The observed differences in electrochemical properties between immature corneocytes and keratinocytes on day 1 indicate the impact of the maturation process. During maturation, the corneous layer experiences dehydration, which directly affects capacitance, dependent on intracellular water content.^[^ ^62^, ^63^ ^]^ This maturation process elucidates the reduction in *C_m,SC_* and the concurrent increase in the *C_m,KL_*/*C_m,SC_* ratio. Subsequently, our analysis highlights the role of the SC layer in safeguarding the KL against dehydration from the air–liquid surface. This explains the observed increase in *C_m,KL_* on day 4, when a robust SC layer is formed, and the subsequent decrease in *C_m,KL_* on day 7, coinciding with the desquamation of the SC layer, as depicted in Figure 2a. These findings highlight the critical importance of considering epidermal keratinocyte differentiation in the context of REE barrier function analysis. To facilitate such assessments, we have devised an equation yielding a parameter for quantifying the contribution of the SC barrier to the REEs, expressed as *R_b,SC_*/(*R_b,KL_* + *R_b,SC_*), as well as the distinct electrochemical charge quantities within the two skin layers, *C_m,KL_*, and *C_m,SC_*. We selected REEs on day 4 as a control group to quantify the effects of chemical and biological dysfunctions within the REEs.

### 2.5 Impedance Analysis of the SDS‐Induced Barrier Dysfunction

**Figure 6:**
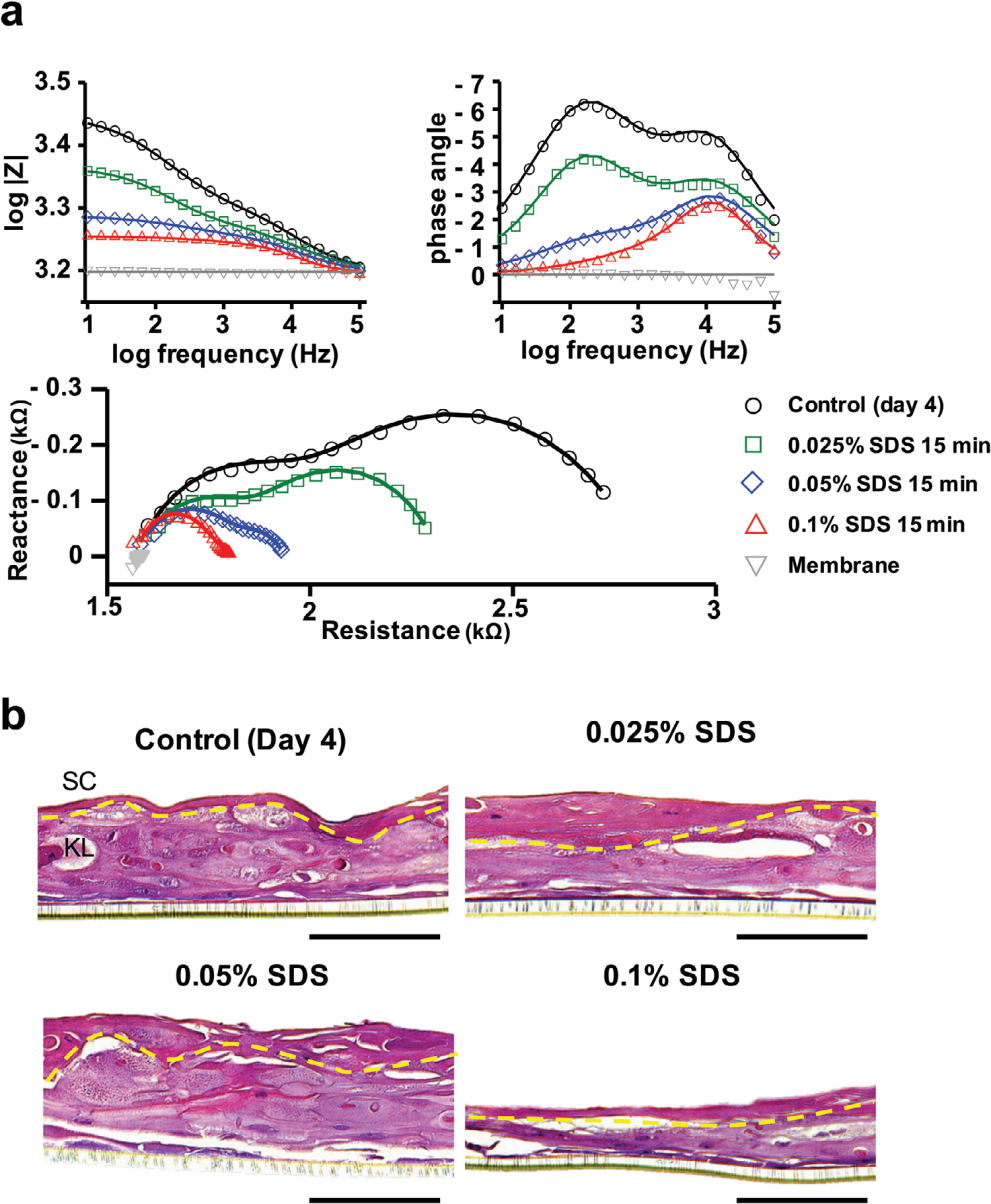
a) The impedance magnitudes (log|Z|), phase angle (θ), and Nyquist plot of the REEs after a 15 min SDS treatment (control, 0.025%, 0.05%, and 0.1%). b) H&E staining image of the REEs after the SDS treatment. Scale bar = 100 µm.

The impedance spectra of REEs after four days of culture, followed by SDS treatment at the air–liquid interface, are shown in **Figure** **6**a. The spectra fit well with the corresponding equivalent circuit model presented in Figure 2c. The impedance spectra declined as SDS concentration increased (control, 0.025%, 0.05%, and 0.1%). The phase angle (PA, θ), a measure of the lag between the alternating current and voltage, reflects the health of cell membranes and mass, with implications for body mass index assessment.^[^ ^64^ ^]^ Ideal capacitors show a 90° voltage lag, indicating full ion storage capacity, which diminishes as capacitors lose their capacity to operate.^[^ ^65^ ^]^ The control REEs exhibited two prominent PA peaks, at 173.7 Hz for the KL and 15,132 Hz for the SC layer, indicative of the plasma membrane capacitance. The absolute PA value at 173.7 Hz decreased significantly from 6.0° to 0.4°, while at 15,132 Hz, it decreased from 5.0° to 4.2° after SDS exposure, signaling a loss of structural and functional integrity in ion storage within the KL's plasma membrane. This structural deterioration is consistent with a substantial decrease in cell viability to 39.3% post 0.1% SDS exposure, as confirmed by the CCK‐8 assay (Figure S6, Supporting Information). Additionally, the H&E‐stained cross‐sectional image of REEs post‐SDS treatment (Figure 6b) revealed that while the SC layer was initially well‐formed after four days of incubation (above the yellow dashed line), increased SDS concentrations resulted in the degradation of both the SC and KL, confirming the irritant nature of SDS.

**Figure 7:**
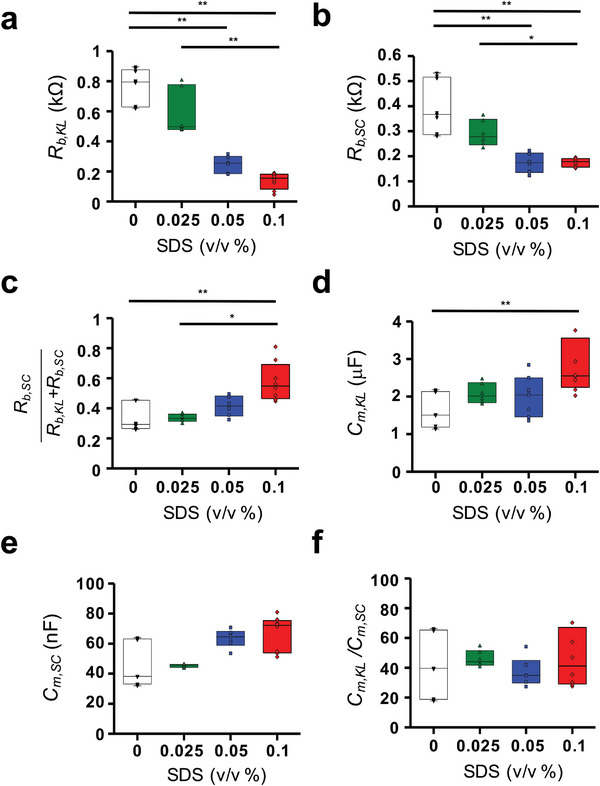
Impedance parameters after treatment with different concentrations of SDS (control, 0.025%, 0.05% and 0.1%).: *R_b,KL_* a), *R_b,SC_* of the REEs b), the electrical contribution using the proposed equation c), *C_m,KL_* d), *C_m,SC_* from Equation (2) e), and *C_m,KL_/C_m,SC_* (f). ^*^ *p *< 0.05 and ^**^ *p *< 0.01.

**Figure** **7** shows the changes in the impedance parameters of the SC and KL layers after performing impedance fitting. Exposure to SDS significantly reduced the barrier resistance of both the SC and KL layers (denoted as *R_b,SC,_* and *R_b,KL_*, respectively). This reduction was more pronounced following treatment with a high SDS concentration (0.1%), with *R_b,KL_* dropping to 17.8% and *R_b,SC_* to 22.8% of their respective control values (Figure 7a,b). Although SDS compromised the integrity of both layers, the extent of barrier disruption varied at elevated SDS concentrations. The ratio *R_b,SC_*/(*R_b,KL_* + *R_b,SC_*) rose from 33.7% to 57.7%, suggesting that the KL layer is more susceptible to damage from SDS treatments, which in turn affects the skin's structural integrity ratio (Figure 7c). However, our method was unable to ascertain the sequential degradation of skin layers by SDS. Compared to the changes observed in resistance values, SDS's impact on the REEs was small in terms of the calculated membrane capacitances of KL and SC (*C_m,KL_* and *C_m,SC_*), as shown in Figure 7d,e. A substantial increase in *C_m,KL_* was observed when a high concentration of SDS was used, which deviated markedly from the control group's results. This increase in capacitance may be attributed to a decrease in the series capacitance caused by the irreversible damage to the cell membranes.^[^ ^35^ ^]^ However, no substantial changes were found in *C_m,SC_*, or the ratio of *C_m,KL_*/*C_m,SC_*. The negligible change in *C_m,SC_* and the considerable variability in the data upon sample exchange likely contributed to this result (Figure 7f).

### 2.6 Time‐Lapse Impedance Measurement of the Barrier Dysfunction Using a Low Concentration of SDS

**Figure 8:**
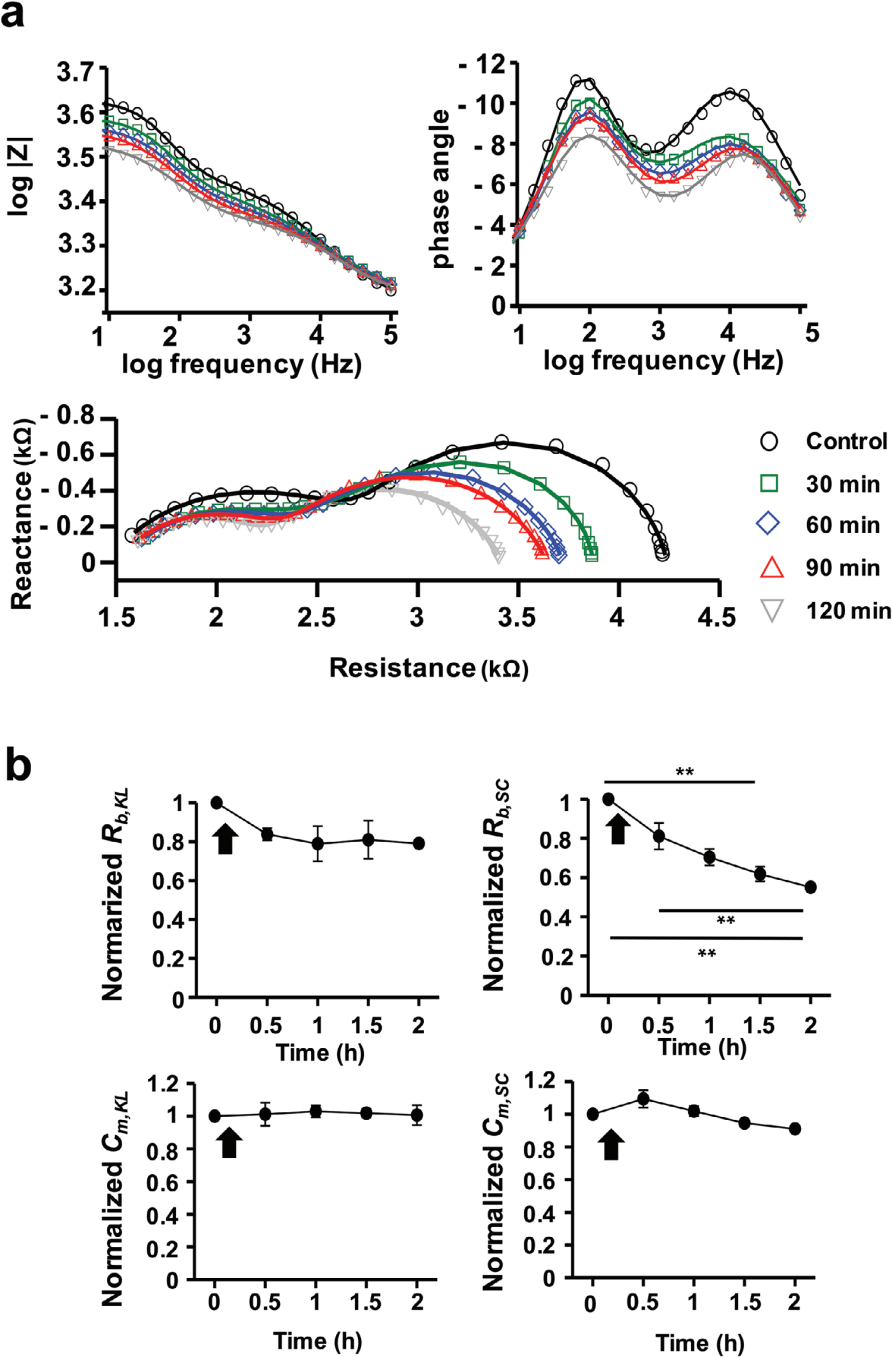
a) The change of the impedance magnitudes (log|Z|), phase angle (θ), and Nyquist plot of the REEs after a 0.025% SDS treatment measured every 30 min. Symbols indicate the measurement data, and curved lines indicate the fitted data. b) The change in normalized impedance parameters over time. The black arrow indicates the time point at which the SDS treatment started.

To reduce sample variability and investigate the effects on the *C_m_* of each skin layer during penetration by a low concentration of SDS, we measured the impedance of the REEs every 30 min following exposure to 0.025% SDS. The impedance decreases as expected (**Figure** **8**a). This trend differed from that presented in Figure 7, indicating distinct patterns of barrier disruption in the KL and SC layers. Specifically, the PA at 15,132 Hz diminished from 10.5° to 6.9°, and at 173.7 Hz, it fell from 10.4° to 8.4°. The non‐zero PA at 173.7 Hz suggests that the cell membranes in the KL still maintain their function as non‐ideal capacitors. Time‐lapse impedance monitoring after the low‐concentration SDS application revealed significant changes in impedance parameters (Figure 8b). Notably, only the normalized *R_b,SC_* experienced a marked decline, implying that SDS micelles predominantly reside within the SC layer. In contrast, the normalized *R_b,KL_* did not show a corresponding reduction. Through impedance analysis, we were able to trace the SDS penetration pathway and the barrier functions of the individual layers over time.

Despite these observations, the calculated *C_m_* values for both layers did not exhibit any change. These are different from the impedance spectra obtained from porcine ear skin (Figure S7A, Supporting Information). Unlike the impedance spectra of the skin equivalents were unable to distinguish between each layer of the skin structure. The skin impedance spectra aligned with the frequency response of a single time constant with one R‐CPE (Figure S7B, Supporting Information). At a higher SDS concentration (0.1%), we noted a decrease in the total resistance of porcine skin (*R_b, porcine skin_*) and an increase in the total membrane capacitance (*C_m, porcine skin_*) (Figure S7C, Supporting Information). Hematoxylin and eosin (H&E) staining confirmed that the extracted porcine skin primarily consisted of the dermis, with the high SDS concentration effectively disrupting the epidermal structure post‐SC penetration (Figure S7D, Supporting Information), corroborating prior studies on SDS‐induced barrier impairment in REEs. An increase in capacitance was attributed to a thinning of the capacitor, predominantly affecting the cell membrane structure of keratinocytes.^[^ ^35^ ^]^ Our findings demonstrate that the applied methodology can differentiate the effects of low irritant concentrations on skin structure and elucidate the hypothesized mechanisms of barrier degradation.

### 2.7 Bioimpedance Simulations of Physiological Changes in Three Possible Models in REEs

**Figure 9:**
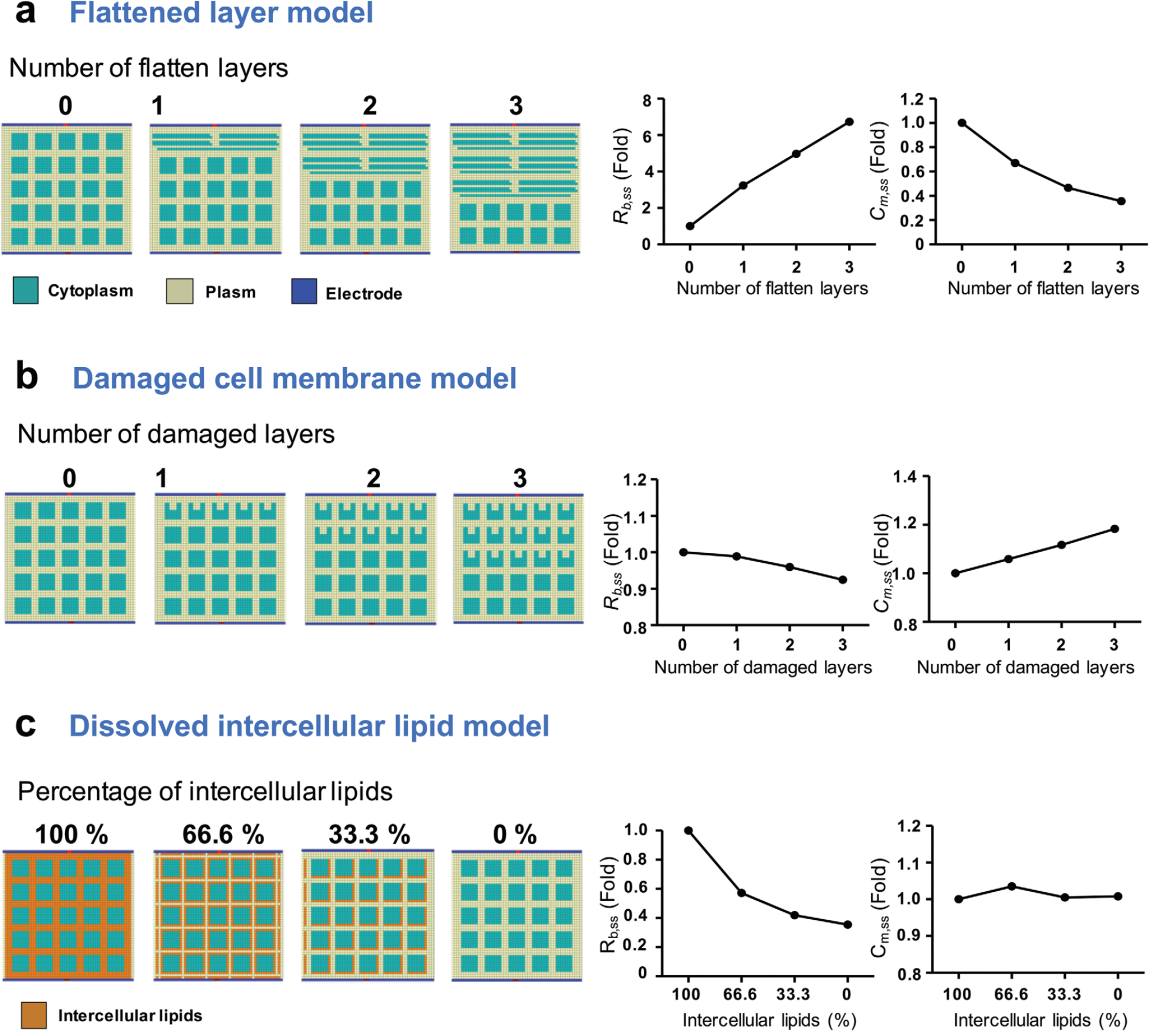
Graphical depiction of possible models of physiological change in an REE: flattened layer model, damaged cell membrane model, and dissolved intercellular lipid. BioZsim simulated parameters (*R_b,ss_* and *C_m,ss_*) obtained from three models: flattened layer model a), damaged cell membrane model b), and dissolved intercellular lipid model c).

The relative fold changes of the circuit parameters – barrier resistance for simulated skin (*R_b,ss_*) and membrane capacitance for simulated skin (*C_m,ss_*) – fitted from a SPICE simulation are shown in **Figure** **9**. We formulated three models to represent potential physiological alterations in skin structure due to maturation or disruption by detergents: the flattened cell layer model, the damaged cell membrane model, and the dissolved intercellular lipid model. The flattened cell layer model reflects the cellular flattening process observed during maturation (Figure 2). Here, *R_b,ss_* increases while *C_m,ss_* decreases in correlation with the number of flattened cell layers (Figure 9a). The simulation indicates that as superficial skin cells become flattened, the impedance parameters are correlated more closely with the changes seen when keratinocytes transition into flattened corneocytes (Figure 5). Notably, the values of *C_m,SC_* are much lower than those of *C_m,KL_*, suggesting that the membrane capacitance decreases as cells flatten. However, the simulation does not account for variations in cell organelles or plasma membrane integrity, limiting the comparative increase of SC barrier resistance to *R_b,KL_*.

Human SC intercellular lipids – comprising primarily fatty acids, cholesterol, cholesteryl sulfate, and sphingolipids – are essential for barrier function and forming multilamellar sheets in intercellular spaces.^[^ ^66^ ^]^ Upon detergent application, the damaged cell membrane model and the dissolved intercellular lipid model can depict structural alterations (Figure 9b,c). *R_b,ss_* decreases in both scenarios, but in the cell membrane‐damaged model, *C_m,ss_* is proportional to the number of damaged layers, which explains the EIS findings on detergent‐induced cell membrane damage (Figure 7; Figure S7, Supporting Information). In contrast, the dissolved intercellular lipid model reflects the time‐lapse impedance measurements of barrier dysfunction with low SDS concentration (Figure 8b), indicating that changes in *C_m_* are driven by morphological alterations in the REEs—such as cell flattening or membrane damage—and are not affected by lipid variations. This suggests that even a limited quantity of SDS micelles can disrupt intercellular lamellar lipids without significantly enlarging the intercellular spaces or harming the plasma membrane.

### 2.8 Impedance Analysis of the Barrier Dysfunction in Psoriasis Skin Model

**Figure 10:**
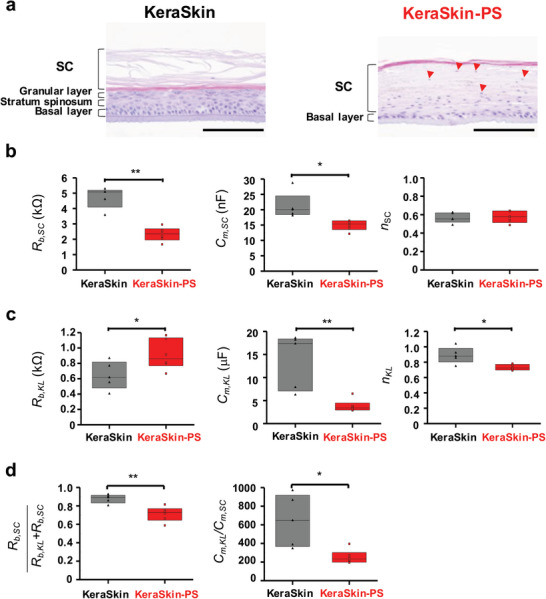
a) H&E images of the control (KeraSkin) and psoriasis model (KeraSkin‐PS). No granular layer can be observed but many preserved nuclei in thick SC (parakeratosis, psoriasiform hyperplasia) are indicated by red arrows. Scale bar = 100 µm. Comparison of the impedance parameters of SC b), KL c) and the contribution ratio between the control REE and psoriasis models d). ^*^ *p* < 0.05 and ^**^ *p* < 0.01 compared to each group.

**Figure** **10**a compares histopathological features between the normal skin model (KeraSkin) and the psoriasis skin model (KeraSkin‐PS). The latter is a commercialized psoriasis REE model that incorporates over‐expressed psoriasis genetic markers (e.g., DEFB4A, S100A7, PI3) and pro‐inflammatory cytokines (e.g., CXCL10, IL‐8). KeraSkin‐PS exhibits characteristic psoriatic changes, such as parakeratosis—abnormal retention of nuclei in the SC indicated by red arrows, psoriasiform hyperplasia, and an abnormally thickened SC layer with a missing granular layer.^[^ ^48^ ^]^

The impedance comparison between KeraSkin and KeraSkin‐PS (Figure 10b–d) reveals a defective barrier function in the psoriasis model. The SC's barrier resistance (*R_b,SC_*) and membrane capacitance (*C_m,SC_*) decrease in KeraSkin‐PS, while the keratinocyte layer's (KL) resistance (*R_b,KL_*) increases, and its capacitance (*C_m,KL_*) significantly reduces. Additionally, the maturation index of KL (*n,_KL_*) decreases, suggesting an overproduction of immature cell layers. This contrasts with the unchanged maturation index of SC (*n,_SC_*), indicating specific impedance parameter alterations in KL but not in SC. The psoriasis model shows a decreased contribution ratio of *R_b,SC_*, and a diminished electrochemical difference between KL and SC, consistent with the recognized barrier dysfunction and immaturity in psoriatic SC.^[^ ^67^ ^]^

Our customized impedance spectroscopy system discriminates physiological changes in the life cycle of keratinocytes and detects barrier dysfunction in disease‐like models. It requires a simplified skin model, like REEs excluding the dermis, to minimize interference effects among layers—complexities observed in the full‐thickness mouse and porcine skin spectra (Figures S4 and S7, Supporting Information). Yet, we demonstrated the electrochemical distinction between fibroblasts in the dermis and KL in full‐thickness models. Our results offer insights into complex human skin models for various diseases and facilitate the development of patient‐derived skin models. Our platform also can help enhance the reproducibility of in vitro skin models, deepen our understanding of physiological changes in disease models, and evaluate the efficacy of targeted therapies. In particular, in conjunction with induced pluripotent stem cell (iPSC) technology, this work has significant potential in providing personalized prognosticative value. iPSC‐derived skin models can be tailored to individual patients, capturing their unique genetic and cellular characteristics.^[^ ^68^, ^69^ ^]^ This enables predictive diagnostics, allowing for early identification of biomarkers associated with itching skin conditions and psoriasis. By integrating non‐invasive EIS, we can detect and quantify early pathological changes, offering real‐time monitoring and personalized treatment plans. Future research should explore the development and validation of iPSC‐derived skin models to fully harness this technology's predictive and diagnostic capabilities.

## 3 Conclusion

In this study, we introduced an impedance spectroscopy approach as a non‐destructive, label‐free, and real‐time monitoring system capable of quantitatively assessing the barrier function of individual layers in the RHE. This method has enabled the observation of two distinct electrochemical barriers corresponding to different maturation stages, facilitating precise measurements of barrier resistance and membrane capacitance. As a result, the integrity of the RHE can be validated before experimental use. Moreover, by applying this technique, we were able to quantify the impact of both chemical and immunological factors on the SC and KL integrity. Specifically, the system allowed us to track physiological changes induced by varying concentrations of SDS and to detect the immature SC formation characteristic of psoriatic skin models. Our system promises to revolutionize quality control processes for RHE production by ensuring consistent barrier functionality and providing a reliable endpoint for assessing the progression of inflammatory skin disease models. Its non‐destructive nature and reduced processing time significantly minimize the risk of false positives. Additionally, the ease of parallelization sets the stage for high‐throughput applications, potentially streamlining drug discovery and toxicology studies. The ability to measure impedance in RHE paves the way for generating robust experimental data across a spectrum of dermatological research. We believe our system will be instrumental in advancing the field by providing accurate, efficient, and reproducible assessments of skin barrier function.

## 4 Experimental Section

### Materials

Sodium dodecyl sulfate (10% v/v, SDS) was obtained from Elpis Biotech (Daejeon, Republic of Korea). Dulbecco's Phosphate‐buffered saline (PBS) was purchased from Welgene (Gyeongsan, Republic of Korea). Dispase II (neutral protease, grade II) was purchased from Roche Diagnostics (Mannheim, Germany).

### In Vitro Human Skin Model

An epidermal equivalent (Neoderm‐E, 24‐well size) was purchased from Tego Science (Seoul, Republic of Korea). A day after the product was received, REEs in the insert were transferred into 24‐well plates on a sterile pad. They were incubated for 4 days or more in a 37 °C, 5% CO_2_ incubator to differentiate keratinocytes into corneocytes in a medium containing 10% fetal bovine serum (FBS) and 1% penicillin. To compare the REE models, a full‐thickness skin model, and skin disease model, an epidermis model (KeraSkin, 24‐well size), a full‐thickness skin model (KeraSkin‐FT, 24‐well sized), and a psoriasis skin model (KeraSkin‐PS, 24‐well sized) were purchased from Biosolution (Seoul, Republic of Korea). The skin models were placed in 24‐well plates filled with KeraSkin‐FT growth medium (0.9 mL) and incubated in a 37 °C, 5% CO_2_ incubator. The medium was changed every two days. KeraSkin‐PS were placed in the well‐plate with KeraSkin‐PS medium for 3 days.

### Ex Vivo Full‐Thickness Mouse Skin and Epidermis Separation

Athymic nude mice (female, 6 weeks old) were purchased from Koatech (Pyeongtaek, Republic of Korea). Samples were extracted from the back skin and cut into small sections (4 × 4 cm). The sample was submerged in a solution of 1 mg mL^−1^ dispase II in PBS for 12 h. The epidermis layer was gently separated from the dermis by surgical tweezers.

### Ex Vivo Full‐Thickness Porcine Skin

Fresh pig skin was purchased from Apures (Pyeongtaek, Republic of Korea) and OptiPharm (Cheongju, Republic of Korea). All porcine skin samples were collected from the ears of pigs. The ear tissue was washed with 70% ethanol and PBS. A punch biopsy was obtained from a flat portion of the ear tissue, and the cartilage in the center was removed to leave only the skin tissue. A 10 mm biopsy was used, the biopsied skin being carefully aligned to the cell culture insert using a porous membrane (Millipore Sigma, Burlington, MA, USA), and a silicone ring of diameter 10 mm was inserted before being placed in a 6‐well plate filled with culture medium. The tissues were pre‐incubated for 24 h at 37 °C in a humidified environment containing 5% CO_2_. The growth medium was replaced every 48 h.

### Cell Viability Assay

A Cell Counting Kit‐8 (CCK‐8, Dojindo, Japan) was used to measure dehydrogenase activity following the manufacturer's instructions. REEs were washed with PBS to remove the media, after which a reagent volume equal to that of each well was added to each well. After 60 min of incubation, absorbance at 450 nm was measured using a Promega GloMax plate reader (Madison, WI, USA).

### Impedance Measurement of Skin Models

The EIS was custom‐manufactured to include upper and lower chambers, in which two gold electrodes were installed. The upper chamber could be moved up and down using a control dial. The system was designed for a 24‐well insert of diameter 10 mm. Two channels with open‐end holes of diameter 4 mm and two separate gold electrodes were vertically interlocked. After the PBS had filled the channel without any bubble generation, the PBS‐filled skin model was placed in the hole of the bottom channel and interlocked in the chamber once again to conduct impedance measurements, the four exposed electrodes being connected to an impedance analyzer (CompactStat, IVIUM, Eindhoven, Netherlands). The impedance was measured over a frequency range of 10 Hz to 100 kHz at five frequencies per decade on a log scale with an amplitude of 200 mV. After measurements were conducted, the insert was replaced with a fresh insert without a cell layer to measure the background impedance. Impedance analysis was performed using the ZView fitting program to determine the impedance parameters.

### Equivalent Circuit Modeling for the REE Model

The system has a PBS and base membrane resistance, *R_pbs_ + R_mem_*, barrier resistance and constant phase element of keratinocyte membrane*, R_b,KL_, CPE_m,KL_*, and barrier resistance and constant phase element of the corneocyte membrane, *R_b,SC_, CPE_m,SC_*, respectively. The constant phase element for the imperfect capacitor, *CPE_m_*, is described as

(1) $$Z_{m}=\frac{1}{CPE_{m}{\left(j\omega \right)}^{n}}$$

where *Z_m_* denotes the impedance value of the cell membrane, *j* denotes the imaginary value, ω denotes the angular frequency, and *n* denotes the secondary parameter of the *CPE*, which is a number between 0 and 1 which indicates the ideality of the capacitor (*n* = 1 for an ideal capacitor). Because of the imperfect capacitive characteristics of the cell membrane, a constant phase element (CPE) can be used for fitting analysis and converted to the calculated membrane capacitance (*C_m_*) using Equation (2) given by Macdonald.^[^ ^70^ ^]^

(2) $$\mathrm{Caculated}\;C_{m}=\frac{{\left(R_{b}\times \mathrm{CP}E_{m}\right)}^{\frac{1}{n}}}{R_{b}}$$

### Impedance Simulation by Changing the Impedance Parameter Using the Equivalent Model for the REE

After the impedance measurement of the 4‐day‐cultured REEs, impedance analysis was performed using ZView fitting program. The skin model parameters *R_b,KL_, CPE_m,KL_*, *R_b,SC_, CPE_m,SC_* were increased or decreased manually, and the simulation results were compared with the fitting results of the 4‐day‐cultured REEs.

### SDS‐Induced Barrier Disruption in the REE

An REE was cultured for four days to differentiate the corneocyte layer using the supplied medium containing 10% FBS. Quantity having 20 µL of different concentrations of SDS (control, 0.025%, 0.05%, and 0.1%) on the air–liquid interface of the REE model for 15 min before rinsing with PBS were treated.

### Impedance Interval Analysis of SDS‐Induced Disruption in Skin Models

The impedance of a single skin model undergoing barrier disruption was measured at constant intervals to remove sample variations. The impedance was measured under the same conditions for the first time. PBS inside the rim was removed using a pipette. SDS (20 µL, 0.025% v/v) was applied to the air–liquid interface for 2 min. The impedance was continuously measured every 30 min for 2 h by applying 200 mV AC over a frequency range of 10 Hz to 100 kHz at five frequencies per decade. The impedance parameters were calculated using impedance spectroscopy and the ZView fitting program.

### Histological Staining

The skin model was taken out of the insert and fixed using 10% formaldehyde. The fixed skin model was dehydrated using a series of graded ethanol from 70 to 100%. The samples were then immersed in xylene. The skin model was placed perpendicular to the mold and filled with paraffin, after which the samples were sectioned into 3 µm thick slices and stained using hematoxylin and eosin (H&E). Bright‐field images were captured using a microscope (DFC450C, Leica Microsystems, Germany).

### BioZsim Simulation

BioZsim, a bioimpedance simulator, was used to estimate the electrical impedance of live tissues undergoing different physiological changes. A user‐drawn 2D mesh of electrical features—including the cytoplasm, plasma, and electrodes—was automatically converted into a simulation program with an integrated circuit emphasis (SPICE) netlist using the BioZsim application. To define the relationship between structural changes in the skin layer and impedance parameters, three potential models of physiological changes in REEs under SDS‐disrupting conditions—that is, a flattened cell layer, a damaged cell membrane, and a dissolved intercellular lipid model were created. All simulations were run on fixed 500 × 500 µm mesh maps, an electrode pair being positioned at the top and bottom. The parameter values used in the simulations are listed in Table S1 (Supporting Information). In the flattened cell‐layer model, differentiated keratinocytes flattened without changing the number of cells; in the damaged cell membrane model, the individual cell volume decreased layer‐by‐layer; finally, the dissolved intercellular lipid model was used to describe how SDS dissolved intercellular lipids without destroying the cell membrane structure while keeping all other variables constant. The single‐dispersion Cole impedance model and the majority of the simulated impedance spectroscopy results exhibited a high correlation. The ZView application was used to examine the changing ratios of the simulated skin barrier resistance (*R_b,ss_*) and cell membrane capacitance of the simulated skin (*C_m,ss_*) after export of the BioZsim data.

### Statistical Analysis

All data are expressed as the mean SD or SEM. The statistical significance of the differences between groups was analyzed utilizing the Student's t‐test or Kruskal–Wallis test using SPSS software (IBM, New York City, NY, USA). Moreover, *p* < 0.05 and *p* < 0.01 imply that the differences between groups were statistically significant.

## Conflict of Interest

The authors declare no conflict of interest.

## Supporting information

Supporting Information

## Data Availability

The data that support the findings of this study are available from the corresponding author upon reasonable request.

## References

[1] Z. Zhang , B. B. Michniak‐Kohn , Pharmaceutics 2012, 4, 26. 24300178 10.3390/pharmaceutics4010026 PMC3834903

[2] M. O. Danso , T. Berkers , A. Mieremet , F. Hausil , J. A. Bouwstra , Exp Dermatol 2015, 24, 48. 25363465 10.1111/exd.12579

[3] R. Neupane , S. H. S. Boddu , J. Renukuntla , R. J. Babu , A. K. Tiwari , Pharmaceutics 2020, 12, 152. 32070011 10.3390/pharmaceutics12020152 PMC7076422

[4] E. Abd , S. A. Yousef , M. N. Pastore , K. Telaprolu , Y. H. Mohammed , S. Namjoshi , J. E. Grice , M. S. Roberts , Clin Pharmacol 2016, 8, 163. 27799831 10.2147/CPAA.S64788 PMC5076797

[5] M. B. Murphrey , J. H. Miao , P. M. Zito , in StatPearls, StatPearls Publishing, Treasure Island (FL) 2023.

[6] S. Zsiko , E. Csanyi , A. Kovacs , M. Budai‐Szucs , A. Gacsi , S. Berko , Sci. Pharm. 2019, 87, 19. 10.3390/pharmaceutics12090803 PMC7559371 32854296

[7] K. M. Jung , S. H. Lee , W. H. Jang , H. S. Jung , Y. Heo , Y. H. Park , S. Bae , K. M. Lim , S. H. Seok , Toxicol. In Vitro 2014, 28, 742. 24625437 10.1016/j.tiv.2014.02.014

[8] B. Srinivasan , A. R. Kolli , M. B. Esch , H. E. Abaci , M. L. Shuler , J. J. Hickman , J Lab Autom 2015, 20, 107. 25586998 10.1177/2211068214561025 PMC4652793

[9] N. Ojeh , B. Akgul , M. Tomic‐Canic , M. Philpott , H. Navsaria , PLoS One 2017, 12, e0174389. 28350869 10.1371/journal.pone.0174389 PMC5370106

[10] Y. S. Lee , S. B. Han , H. J. Ham , J. H. Park , J. S. Lee , D. Y. Hwang , Y. S. Jung , D. Y. Yoon , J. T. Hong , J. Allergy Clin. Immunol. 2020, 146, 156. 31931018 10.1016/j.jaci.2019.12.905

[11] M. Schafer‐Korting , U. Bock , W. Diembeck , H. J. Dusing , A. Gamer , E. Haltner‐Ukomadu , C. Hoffmann , M. Kaca , H. Kamp , S. Kersen , M. Kietzmann , H. C. Korting , H. U. Krachter , C. M. Lehr , M. Liebsch , A. Mehling , C. Muller‐Goymann , F. Netzlaff , F. Niedorf , M. K. Rubbelke , U. Schafer , E. Schmidt , S. Schreiber , H. Spielmann , A. Vuia , M. Weimer , Altern. Lab. Anim. 2008, 36, 161. 18522484 10.1177/026119290803600207

[12] H. Yousef , M. Alhajj , S. Sharma , in StatPearls, StatPearls Publishing, Treasure Island (FL) 2023.

[13] S. Mitragotri , J. Control Release 2003, 86, 69. 12490374 10.1016/s0168-3659(02)00321-8

[14] Y. Yang , S. Sunoqrot , C. Stowell , J. Ji , C. W. Lee , J. W. Kim , S. A. Khan , S. Hong , Biomacromolecules 2012, 13, 2154. 22621160 10.1021/bm300545b PMC3392468

[15] K. H. Lee , K. A. Cho , J. Y. Kim , J. Y. Kim , J. H. Baek , S. Y. Woo , J. W. Kim , Exp. Dermatol. 2011, 20, 149. 21255094 10.1111/j.1600-0625.2010.01203.x

[16] W. Choi , H.‐C. Shin , J. M. Kim , J.‐Y. Choi , W.‐S. Yoon , J. Electrochem. Sci. Technol. 2020, 11, 1.

[17] J. M. Kim , J. H. Ji , Y. S. Kim , S. Lee , S. Y. Oh , S. J. Huh , C. H. Son , J. H. Kang , S. Y. Ahn , J. E. Choo , K. H. Song , M. S. Roh , Cancers (Basel) 2020, 12, 3120. 33113881 10.3390/cancers12113120 PMC7692663

[18] M. Suarez‐Farinas , S. J. Tintle , A. Shemer , A. Chiricozzi , K. Nograles , I. Cardinale , S. Duan , A. M. Bowcock , J. G. Krueger , E. Guttman‐Yassky , J. Allergy Clin. Immunol. 2011, 127, 954. 21388663 10.1016/j.jaci.2010.12.1124 PMC3128983

[19] T. Montero‐Vilchez , M. V. Segura‐Fernandez‐Nogueras , I. Perez‐Rodriguez , M. Soler‐Gongora , A. Martinez‐Lopez , A. Fernandez‐Gonzalez , A. Molina‐Leyva , S. Arias‐Santiago , J Clin Med. 2021, 10, 359. 33477944 10.3390/jcm10020359 PMC7833436

[20] M. J. Cork , S. G. Danby , Y. Vasilopoulos , J. Hadgraft , M. E. Lane , M. Moustafa , R. H. Guy , A. L. Macgowan , R. Tazi‐Ahnini , S. J. Ward , J. Invest. Dermatol. 2009, 129, 1892. 19494826 10.1038/jid.2009.133

[21] K. Heinrich , U. Heinrich , H. Tronnier , Skin Pharmacol Physiol 2014, 27, 141. 24434680 10.1159/000354919

[22] J. Logger , J. Olydam , W. Woliner‐van der Weg , P. van Erp , Cosmetics 2019, 6, 20.

[23] Zsikó, C. , Kovács, S. , Budai, G. , Berkó , Sci. Pharm. 2019, 87, 19.

[24] C. Gorzelanny , C. Mess , S. W. Schneider , V. Huck , J. M. Brandner , Pharmaceutics 2020, 12, 684. 32698388 10.3390/pharmaceutics12070684 PMC7407329

[25] V. van Drongelen , M. O. Danso , A. Mulder , A. Mieremet , J. van Smeden , J. A. Bouwstra , A. El Ghalbzouri , Tissue Eng Part A. 2014, 20, 3041. 24819925 10.1089/ten.tea.2014.0011 PMC4229711

[26] C. Mathes , J. M. Brandner , M. Laue , S. S. Raesch , S. Hansen , A. V. Failla , S. Vidal , I. Moll , U. F. Schaefer , C. M. Lehr , Eur. J. Cell Biol. 2016, 95, 89. 26785612 10.1016/j.ejcb.2015.12.001

[27] C. Choe , J. Ri , J. Schleusener , J. Lademann , M. E. Darvin , Exp Dermatol 2019, 28, 1237. 31400168 10.1111/exd.14018

[28] K. Derr , J. Zou , K. Luo , M. J. Song , G. S. Sittampalam , C. Zhou , S. Michael , M. Ferrer , P. Derr , Tissue Eng Part C Methods 2019, 25, 334. 31007132 10.1089/ten.tec.2018.0318 PMC6589501

[29] R. Pouliot , L. Germain , F. A. Auger , N. Tremblay , J. Juhasz , Biochim. Biophys. Acta 1999, 1439, 341. 10446422 10.1016/s1388-1981(99)00086-4

[30] Y. Dancik , G. Sriram , B. Rout , Y. Zou , M. Bigliardi‐Qi , P. L. Bigliardi , Analyst 2018, 143, 1065. 29368763 10.1039/c7an01675a

[31] C. Choe , J. Schleusener , S. Choe , J. Lademann , M. E. Darvin , J Biophotonics 2020, 13, e201960106. 31602797 10.1002/jbio.201960106

[32] T. Yamamoto , Y. Yamamoto , Med Biol Eng 1976, 14, 494. 979374 10.1007/BF02478045

[33] A. H. Gitter , J. D. Schulzke , D. Sorgenfrei , M. Fromm , J. Biochem. Biophys. Methods 1997, 35, 81. 9350514 10.1016/s0165-022x(97)00028-6

[34] L. J. Gentet , G. J. Stuart , J. D. Clements , Biophys. J. 2000, 79, 314. 10866957 10.1016/S0006-3495(00)76293-X PMC1300935

[35] F. Groeber , L. Engelhardt , S. Egger , H. Werthmann , M. Monaghan , H. Walles , J. Hansmann , Pharm. Res. 2015, 32, 1845. 25467957 10.1007/s11095-014-1580-3 PMC4381093

[36] M. Tatullo , M. Marrelli , M. Amantea , F. Paduano , L. Santacroce , S. Gentile , S. Scacco , J Cancer 2015, 6, 976. 26366210 10.7150/jca.11936 PMC4565846

[37] F. Lu , C. Wang , R. Zhao , L. Du , Z. Fang , X. Guo , Z. Zhao , Biosensors (Basel) 2018, 8, 31. 29587456 10.3390/bios8020031 PMC6023082

[38] S. Grimnes , Ø. G. Martinsen , Ø. G. Martinsen , Bioimpedance and Bioelectricity Basics, Elsevier/Academic Press, Amsterdam 2015.

[39] E. Hernandez‐Balaguera , H. Vara , J. L. Polo , J. Electrochem. Soc. 2018, 165, G3104.

[40] S. Bjorklund , T. Ruzgas , A. Nowacka , I. Dahi , D. Topgaard , E. Sparr , J. Engblom , Biophys. J. 2013, 104, 2639. 23790372 10.1016/j.bpj.2013.05.008 PMC3686338

[41] L. Kiesewetter , L. Littau , H. Walles , A. R. Boccaccini , F. Groeber‐Becker , Biosens. Bioelectron. 2019, 142, 111555. 31408825 10.1016/j.bios.2019.111555

[42] L. S. Beier , A. Waldow , S. Khomeijani Farahani , R. Mannweiler , S. Vidal‐Y‐Sy , J. M. Brandner , J. Piontek , D. Günzel , Ann. N. Y. Acad. Sci. 2022, 1517, 251. 35994210 10.1111/nyas.14879

[43] A. O. Rinaldi , H. Morita , P. Wawrzyniak , A. Dreher , S. Grant , P. Svedenhag , C. A. Akdis , Allergy 2019, 74, 1934. 30989659 10.1111/all.13824

[44] A. O. Rinaldi , A. Korsfeldt , S. Ward , D. Burla , A. Dreher , M. Gautschi , B. Stolpe , G. Tan , E. Bersuch , D. Melin , N. Askary Lord , S. Grant , P. Svedenhag , K. Tsekova , P. Schmid‐Grendelmeier , M. Möhrenschlager , E. D. Renner , C. A. Akdis , Allergy 2021, 76, 3066. 33830511 10.1111/all.14842

[45] R. Mannweiler , S. Bergmann , Y. S. S. Vidal , J. M. Brandner , D. Gunzel , Allergy 2021, 76, 3094. 33844311 10.1111/all.14851

[46] J. Volkmann , N. Klitzsch , J Appl Geophys 2015, 114, 191.

[47] D. Miklavčič , N. Pavšelj , F. X. Hart , *In* Wiley Encyclopedia of Biomedical Engineering, (Eds.: M. Akay ), Wiley‐Interscienc, Hoboken, New Jersey 2006.

[48] S. K. Raychaudhuri , E. Maverakis , S. P. Raychaudhuri , Autoimmun Rev. 2014, 13, 490. 24434359 10.1016/j.autrev.2014.01.008

[49] B. E. Kim , D. Y. M. Leung , Allergy Asthma Immunol. Res. 2018, 10, 207. 29676067 10.4168/aair.2018.10.3.207 PMC5911439

[50] K. A. Cho , J. Y. Kim , S. Y. Woo , H. J. Park , K. H. Lee , C. U. Pae , Ann. Dermatol. 2012, 24, 398. 23197904 10.5021/ad.2012.24.4.398 PMC3505769

[51] W. J. Shin , Y. K. Kim , K. H. Lee , B. L. Seong , Biosci Biotechnol Biochem 2012, 76, 581. 22451404 10.1271/bbb.110764

[52] H. S. Seo , K. H. Seong , C. D. Kim , S. J. Seo , B. C. Park , M. H. Kim , S. P. Hong , Ann Dermatol 2019, 31, 186. 33911567 10.5021/ad.2019.31.2.186 PMC7992668

[53] G. E. Pierard , V. Goffin , T. Hermanns‐Le , C. Pierard‐Franchimont , Int J Mol Med 2000, 6, 217. 10891569 10.3892/ijmm.6.2.217

[54] M. I. Koster , Ann. N. Y. Acad. Sci. 2009, 1170, 7. 19686098 10.1111/j.1749-6632.2009.04363.x PMC2861991

[55] T. J. Freeborn , B. Maundy , A. S. Elwakil , Med. Biol. Eng. Comput. 2014, 52, 749. 25023892 10.1007/s11517-014-1175-5

[56] N. J. Starr , M. H. Khan , M. K. Edney , G. F. Trindade , S. Kern , A. Pirkl , M. Kleine‐Boymann , C. Elms , M. M. O'Mahony , M. Bell , M. R. Alexander , D. J. Scurr , Proc Natl Acad Sci U S A 2022, 119, e2114380119. 35298332 10.1073/pnas.2114380119 PMC8944899

[57] M. Egawa , H. Tagami , Br J. Dermatol 2008, 158, 251. 18047517 10.1111/j.1365-2133.2007.08311.x

[58] D. Antonov , S. Schliemann , P. Elsner , Curr Probl Dermatol 2016, 49, 61. 26844898 10.1159/000441546

[59] Y. Guo , J. R. Dreier , J. Cao , H. Du , S. R. Granter , D. J. Kwiatkowski , PLoS One 2016, 11, e0167384. 27907099 10.1371/journal.pone.0167384 PMC5132223

[60] T. Gratieri , Y. N. Kalia , Adv Drug Deliv Rev. 2013, 65, 315. 22626977 10.1016/j.addr.2012.04.012

[61] V. Manouras , S. Stathopoulos , A. Serb , T. Prodromakis , Sci. Rep. 2021, 11, 20599. 34663849 10.1038/s41598-021-00001-6 PMC8523686

[62] L. Eckhart , S. Lippens , E. Tschachler , W. Declercq , Biochim. Biophys. Acta. 2013, 1833, 3471. 23792051 10.1016/j.bbamcr.2013.06.010

[63] T. Matsumoto , H. Yuasa , R. Kai , H. Ueda , S. Ogura , Y. Honda , J. Dermatol. 2007, 34, 447. 17584321 10.1111/j.1346-8138.2007.00308.x

[64] S. Kumar , A. Dutt , S. Hemraj , S. Bhat , Manipadybhima B. , Iran J. Basic Med. Sci. 2012, 15, 1180,. 23653848 PMC3646229

[65] T. Islam , L. Kumar , S. A. Khan , Sens. Actuators B Chem. 2012, 173, 377.

[66] K. Harada , T. Murakami , N. Yata , S. Yamamoto , J. Invest. Dermatol. 1992, 99, 278. 1512463 10.1111/1523-1747.ep12616623

[67] S. J. Brown , M. S. Elias , M. Bradley , Acta. Derm. Venereol. 2020, 100, adv00163. 32412647 10.2340/00015555-3513 PMC9189740

[68] M. Itoh , N. Umegaki‐Arao , Z. Guo , L. Liu , C. A. Higgins , A. M. Christiano , PLoS One 2013, 8, e77673. 24147053 10.1371/journal.pone.0077673 PMC3795682

[69] P. Khurana , N. Kolundzic , C. Flohr , D. Ilic , Exp. Dermatol 2021, 30, 1572. 33864704 10.1111/exd.14358

[70] J. R. Macdonald , Solid State Ion 1984, 13, 147.

