# Supplementary material for: Assessing Barrier Function in Psoriasis and Cornification Models of Artificial Skin Using Non‐Invasive Impedance Spectroscopy
Source: Adv Sci (Weinh). 2024 Jul 12;11(34):2400111. doi: 10.1002/advs.202400111 (PMC11575500; doi:10.1002/advs.202400111)
Supplement: Supplementary file 1 — Supporting Information [file ADVS-11-2400111-s001.docx]

## Supporting Information

**Assessing Barrier Function in Psoriasis and Cornification Models of Artificial Skin Using Non-invasive Impedance Spectroscopy**

*Jaehwan Ahn and Yoon Sung Nam^*^*

Dr. J. Ahn and Prof. Y. S. Nam

Department of Materials Science and Engineering, Korea Advanced Institute of Science and Technology, 291 Daehak-ro, Yuseong-gu, Daejeon, 34141, Republic of Korea

E-mail: yoonsung@kaist.ac.kr (Y.S.N.)

Prof. Y. S. Nam

Department of Biological Sciences, Korea Advanced Institute of Science and Technology, 291 Daehak-ro Yuseong-gu, Daejeon, 34141, Republic of Korea

E-mail: yoonsung@kaist.ac.kr (Y.S.N.)

Table S1. Geometric parameters based on the number of channels employed in the bioimpedance simulator (BioZsim) for the passive electrical components of REEs

| **Basic parameters** | | | **Value** | | | | | | |
| --- | --- | --- | --- | --- | --- | --- | --- | --- | --- |
| Pixel size | | | 1 μm^2^ | | | | | | |
| Thickness | | | 50 μm | | | | | | |
| Membrane capacitance | | | 1 μF cm^-2^ | | | | | | |
| Membrane resistance | | | 1 GΩ cm^2^ | | | | | | |
| Plasm resistivity | | | 100 Ω cm | | | | | | |
| **Specific parameters** | | **Flattened layer model** | | | | | | | |
| Number of flatten layer | | | 0 | 1 | | 2 | | 3 | |
| Normal cell size | | | 49 μm^2^ | | | | | | |
| Flatten cell size | | | 49 μm^2^ | | | | | | |
| Cytoplasm resistivity | | | 100 Ω cm | | | | | | |
| **Specific parameters** | **Damaged cell membrane model** | | | | | | | | |
| Number of flatten layer | | | 0 | | 1 | | 2 | | 3 |
| Normal cell size | | | 49 μm^2^ | | | | | | |
| Damaged cell size | | | 40 μm^2^ | | | | | | |
| Cytoplasm resistivity | | | 100 Ω cm | | | | | | |
| **Specific parameters** | **Dissolved intercellular lipid model** | | | | | | | | |
| Dissolved intercellular lipid (%) | | | 0 % | 33 % | | 66 % | | 100 % | |
| Dissolved intercellular lipid area | | | 1,691 μm^2^ | 1,127 μm^2^ | | 563 μm^2^ | | 0 μm^2^ | |
| Total intercellular lipid area | | | 2,916 μm^2^ | 2,916 μm^2^ | | 2,916 μm^2^ | | 2,916 μm^2^ | |
| Intercellular lipid resistance | | | 10 Ω cm^2^ | | | | | | |
| Individual cell size | | | 49 μm^2^ | | | | | | |
| Number of cells | | | 25 | | | | | | |
| Cytoplasm resistivity | | | 100 Ω cm | | | | | | |


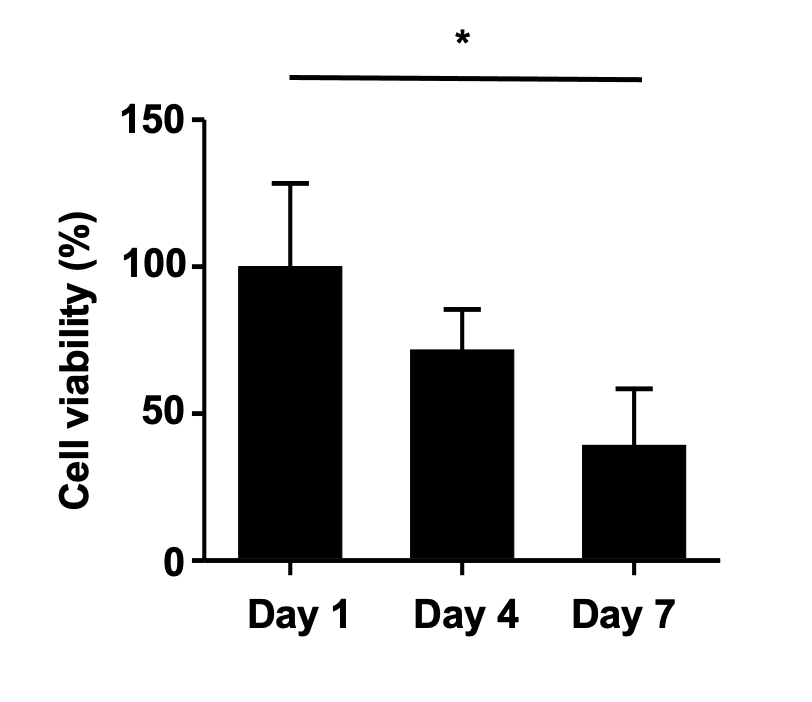


Figure S1. Cell viability of the REEs on days 1, 4, and 7 of incubation. ^*^*p* < 0.05 compared to each group.


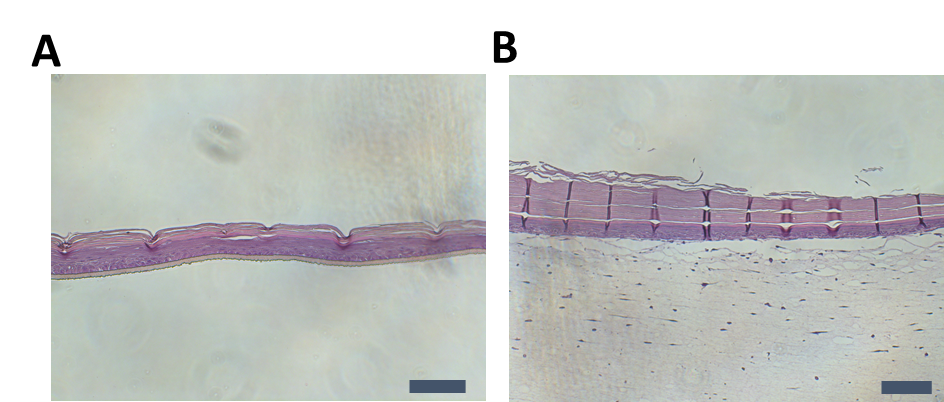


Figure S2. H&E staining image of the (A) REEs (KeraSkin) and (B) full-thickness skin model (KeraSkin-FT) on day 4. KeraSkin-FT has an extra dermis layer consisting of fibroblasts in a collagen matrix. Scale bar = 100 μm.


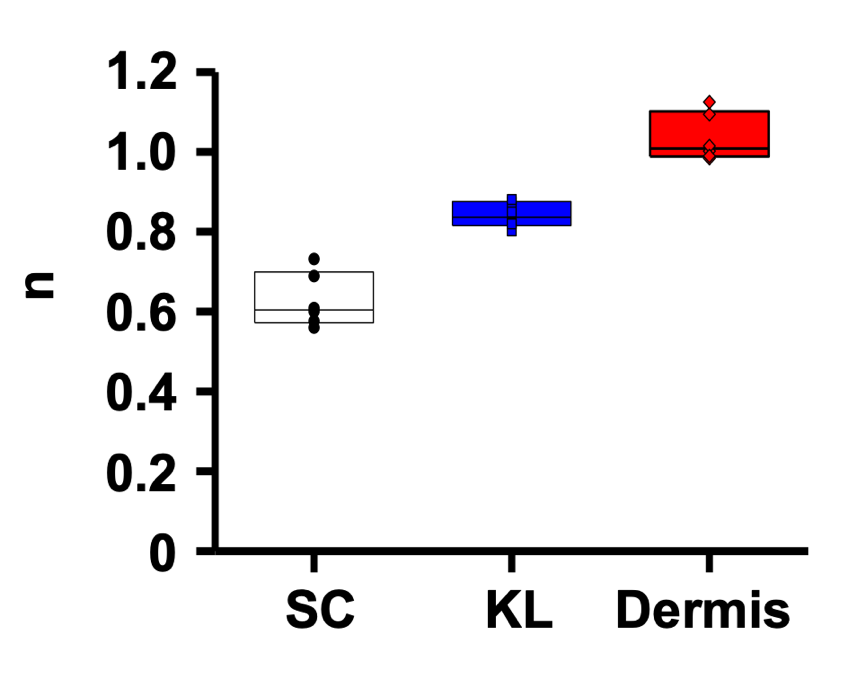


Figure S3. Secondary value of *CPE*, *n* of three skin cell layers in KeraSkin-FT.


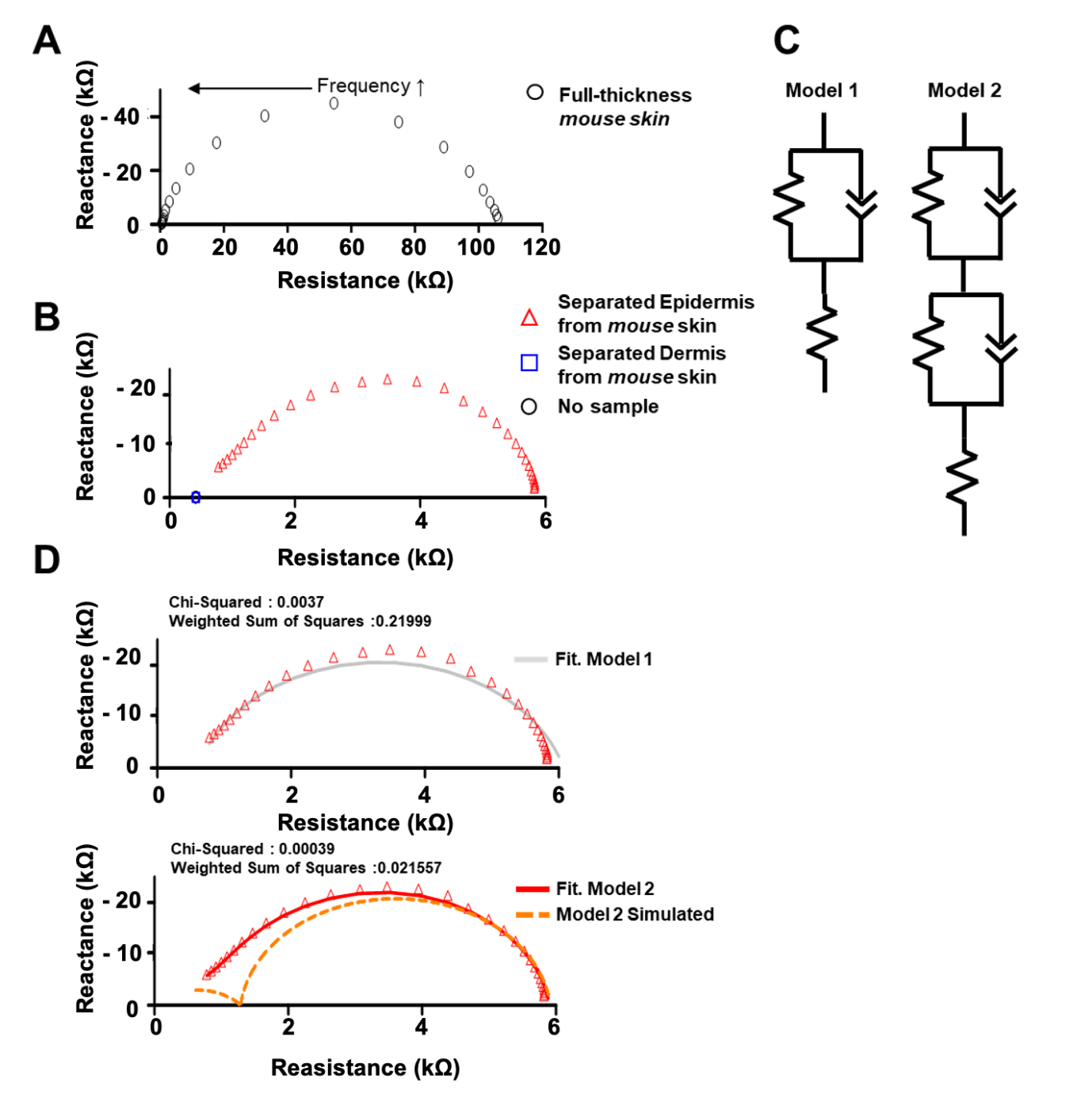


Figure S4. Nyquist plots of the full-thickness mouse (A) and dispase II-separated epidermis and dermis (B) with the frequency range of 10 Hz to 10^5^ Hz. (C) Two equivalent models (model 1 and model 2) were used for the fitting analysis of extracted epidermis layer. (D) Nyquist plots of the separated epidermis (B), where symbols indicate the measurement data, curved lines indicate the fitted data, and orange dot lines indicate the simulated contributions of electrochemical barriers in model 2. Also, the values of the goodness of fitting (chi-squared and weighted sum of squares) were inserted above the data.


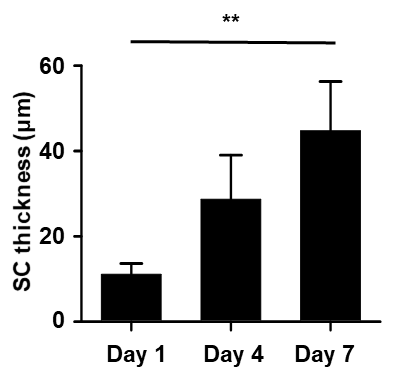


Figure S5. The thickness of the SC layer of the REEs on days 1, 4, and 7 of incubation. *^**^p* < 0.01 compared to each group.


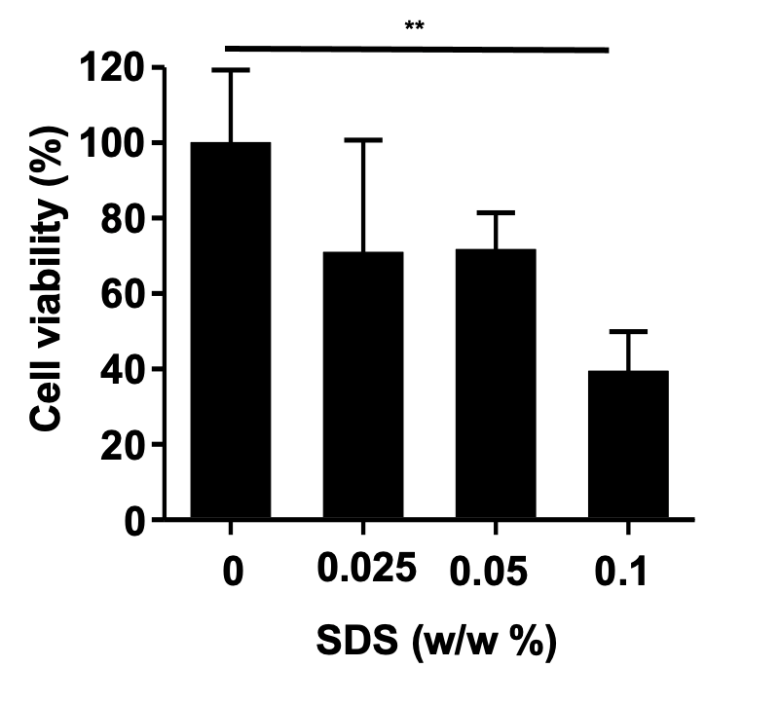


Figure S6. Cell viability of the REEs for different concentrations (control, 0.025 %, 0.05 %, and 0.1 %) of the SDS treatment. *^**^p* < 0.01.


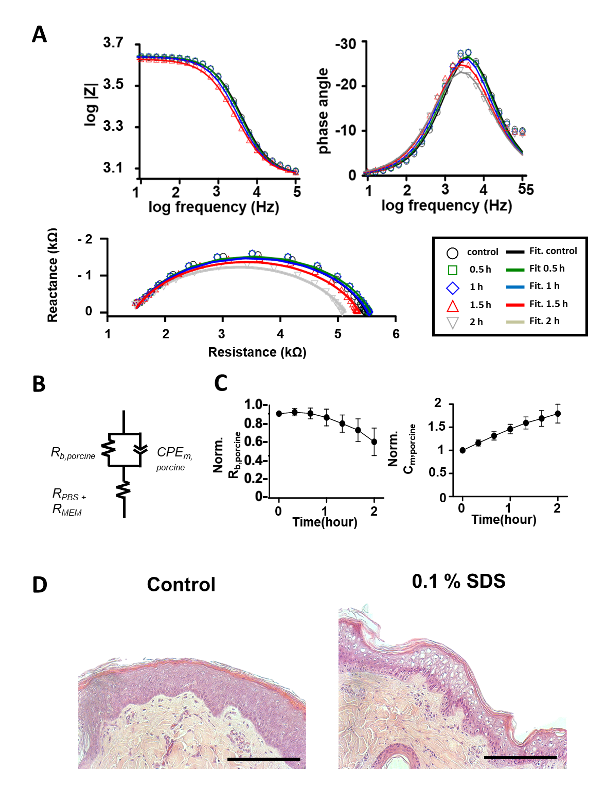


Figure S7. (A) The change of the impedance magnitudes (log|Z|), phase angle (θ), and Nyquist plot of the porcine skin after a 0.1 % SDS treatment measured every 30 min. Symbols indicate the measurement data and curved lines indicate the fitted data. (B) shows the equivalent circuit model for the porcine ear. (C) The time-dependent change of the normalized impedance parameters every 20 min. (D) H&E images of the porcine skin after the 0.1 % SDS treatment for 2 h. Scale bar = 100 μm.
